# Awake perception is associated with dedicated neuronal assemblies in the cerebral cortex

**DOI:** 10.1038/s41593-022-01168-5

**Published:** 2022-09-28

**Authors:** Anton Filipchuk, Joanna Schwenkgrub, Alain Destexhe, Brice Bathellier

**Affiliations:** 1 Paris-Saclay University, CNRS, Paris-Saclay Institute of Neuroscience, Saclay, France; 2 Institut Pasteur, Université de Paris, INSERM, Institut de l’Audition, Paris, France; 3 Present Address: Healthy Mind, Institut du Cerveau - ICM, Paris, France

**Keywords:** Cortex, Sensory processing

## Abstract

Neural activity in the sensory cortex combines stimulus responses and ongoing activity, but it remains unclear whether these reflect the same underlying dynamics or separate processes. In the present study, we show in mice that, during wakefulness, the neuronal assemblies evoked by sounds in the auditory cortex and thalamus are specific to the stimulus and distinct from the assemblies observed in ongoing activity. By contrast, under three different anesthetics, evoked assemblies are indistinguishable from ongoing assemblies in the cortex. However, they remain distinct in the thalamus. A strong remapping of sensory responses accompanies this dynamic state change produced by anesthesia. Together, these results show that the awake cortex engages dedicated neuronal assemblies in response to sensory inputs, which we suggest is a network correlate of sensory perception.

## Abstract (web summary)

Filipchuk et al. show that when awake mice perceive sounds, the auditory cortex produces sound-specific neuronal assemblies distinct from its ongoing activity, whereas under anesthesia sound-evoked assemblies are indistinguishable from ongoing activity.

## Main

It has long been noticed that the circuits of sensory areas in the cerebral cortex display intense ongoing activity in the absence of stimuli from their dedicated sensory modality^1^. The role of this ongoing activity and its relationship to evoked sensory responses remain unclear. Initial observations made under anesthesia in the visual cortex of several mammals^2–4^ have shown a striking similarity between ongoing activity patterns on the mesoscopic scale and sensory responses, suggesting that ongoing activity could be a form of replay of sensory responses. Similar results have been obtained in rat^5,6^ and guinea-pig^7^ primary auditory cortex.

However, recently, recordings in the visual cortex of awake mice have shown that ongoing cortical activity in wakefulness is highly correlated to both the level of arousal^8,9^ and the animal’s facial motor activity patterns^10,11^. Arousal-related fluctuations are also seen in the visual thalamus^12^. Although the direction of causality between behavioral and cortical observables remains to be established, this suggests that ongoing cortical dynamics in the awake state is more than a replay of past sensory activity. In line with this, it was also observed that, even if ongoing and evoked activity recruit similar sets of neurons, they correspond to activity patterns that live in orthogonal neuronal dimensions^10,13^.

These conflicting observations across physiological states suggest that anesthesia triggers a profound transformation of neuronal dynamics in the cortical circuits and beyond^14^. This idea is supported by strong effects of anesthesia on neuronal integration in cortical neurons^15^, but also on corticocortical and to a lesser extent thalamocortical connections^16,17^. Field potential recordings and single-cell analysis of cortical neurons across wakefulness and anesthesia indicate modulations of responsiveness to sounds and decreased signal:noise ratio under anesthesia^18,19^. However, these studies do not provide spatially resolved information about thalamocortical population activity patterns, which prevents the determination of whether similarity of evoked and spontaneous population patterns is an effect of anesthesia or a more generic phenomenon.

In the present study, we imaged ongoing and sound-evoked activity in large populations of mouse auditory cortex neurons, as well as axonal terminals from the auditory thalamus, across wakefulness and three different types of anesthesia. We observed that the cortex generates distinct evoked and ongoing cell assemblies during wakefulness, which supports an accurate encoding of diverse sounds. In contrast, under anesthesia, ongoing and evoked activity patterns became indistinguishable. As a consequence, despite the presence of specific sound responses in the anesthetized state, sound representations were strongly impoverished and markedly different to the representations observed in the awake state. In thalamocortical axons, we observed distinct ongoing and evoked assemblies in wakefulness, and in the present study, the two types of assemblies remained different under anesthesia. This indicates a functional disconnection between cortex and its thalamic inputs under anesthesia, whereas the existence of distinct sound-specific and ongoing cortical cell assemblies seems to be a signature of awake perception.

## Results

### Population events under anesthesia and in wakefulness

**Fig. 1:**
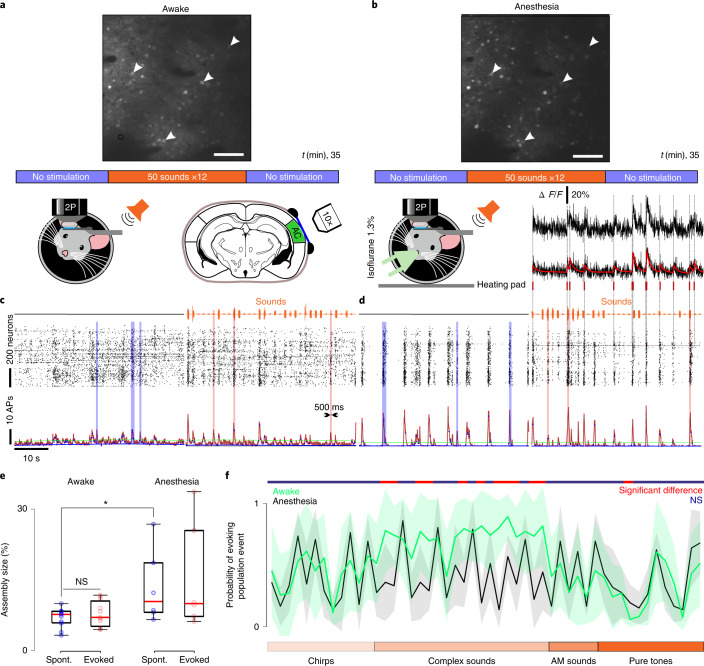
Synchronous population events in the auditory cortex in wakefulness and anesthesia. **a**, Two-photon Ca^2+^ imaging at a 30-Hz sampling rate of up to 1,200 layer 2/3 neurons expressing GCaMP6s in the awake head-fixed mouse. Upper: imaging field of view with labeled neurons in the awake state showing the s.d. of fluorescence pixels over a 15-min sound stimulation session flanked by two 10-min-long stimulation-free periods. Scale bar, 100 μm. **b**, The same recording protocol and field of view as in **a**, but under light isoflurane anesthesia (1.3%). Most neurons are visible in both conditions, demonstrating full stability of the field of view. Weaker or absent labeling in anesthesia reflects neurons that have decreased their activity. Arrows indicate sample neurons. Scale bar, 100 μm. Bottom right: spike time estimates (red) were extracted from calcium fluorescence traces (black) using the MLSpike algorithm. **c**, Population raster plots and population firing rate (30-ms bins) during no stimulation (left) and stimulation (right) periods. Vertical transparent bars highlight spontaneous (blue) and evoked (pink) population events detected as described in Extended Data Fig. 1d. **d**, Same as in **c** but for anesthesia. **e**, The size of ongoing population events slightly increases under anesthesia (*P* = 0.036, *n* = 11 for awake and *n* = 6 for anesthesia, *P* = 0.18 for evoked events' Wilcoxon’s rank-sum test). In the box-and-whisker plots, the red mark indicates the median and the bottom and top edges of the box indicate the 25th and 75th percentiles, respectively. The whiskers extend to the extreme data points. **f**, Probability of sounds evoking population events in the awake state (green trace) and under anesthesia (black trace). The shaded areas indicate the s.d. around the mean. Most of the complex sounds significantly decrease their ability to drive population events (*P* < 0.05) when passing from wakefulness to anesthesia (Wilcoxon’s rank-sum test for **e** and **f**). AM, amplitude modulated; Spont., spontaneous; *P < 0.05; NS, not significant. All tests are two sided. Source data

**Extended Data Fig. 1:**
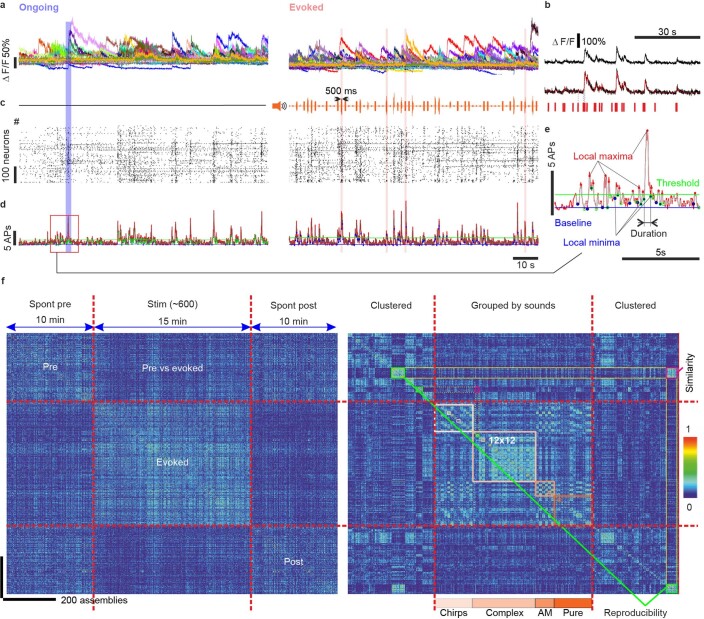
Identification and clustering of spontaneous and evoked assemblies. **a**, Examples of raw calcium fluorescence traces without stimulation (left) and during sound stimulation (right). **b**, Spikes time estimates (red) were extracted from calcium fluorescence traces (top trace: example raw signal) using the MLSpike algorithm (bottom trace: the raw fluorescent trace adjusted to the baseline fluorescence fluctuations). **c**, Population raster plots during no stimulation (left) and stimulation (right) periods. Every dot represents a spike estimated as in **b**. **d**, Population firing rate (30 ms bins) during no stimulation (left) and stimulation (right) periods. Vertical transparent bars highlight spontaneous (blue) and evoked (pink) population events detected as described in **e** (inset). **e**, Identification of spontaneous population events from population firing rate (red trace). Stochastic threshold (green), based on population spiking activity and added to the baseline (blue), to identify the local maxima above the threshold (red dots) in the smoothed population firing rate trace (gray). Local minima (blue and green dots) adjacent to the above threshold local maxima define the beginning and the end of the population event, respectively. All neurons spiking at least once within this interval are included into the population event, defining spontaneous or evoked neural assemblies. **f**, Left panel: For an example recording session, Pearson’s correlation matrix between spontaneous assemblies (pre- and post-stimulation) and the assemblies evoked by a single trial sound response (50 sounds presented 12 times each) in the order of their appearance during 35-minute recording in awake mouse. Diagonal quadrants show the auto-correlation between the same types of assemblies, lateral quadrants – cross-correlation between different types of assemblies. Right panel: for the same data as in the left panel, similarity matrix in which spontaneous assemblies are sorted by hierarchical clustering and evoked responses (whether or not a population event was detected) are sorted sound-by-sound (12 trials/sound). The mean correlation among the assemblies in a cluster or among the assemblies corresponding to single trial sound responses define the reproducibility of a spontaneous or evoked pattern (green inset). The mean cross-correlation between two groups of patterns defines their similarity (pink inset). Color scale for pattern correlation values (red for 1, dark blue for 0). (AM, amplitude modulated sounds; Pure, Pure tones; Spont, spontaneous; Stim, Stimulation).

Taking advantage of the robustness of GCaMP6s-based^20^, two-photon calcium imaging for the assignment of neuronal activity in identified neurons, we contrasted neural population activity in the auditory cortex of head-fixed mice, in the awake state (Fig. 1a) and during light isoflurane anesthesia (Fig. 1b). A first 10-min imaging session without auditory stimuli was followed by a 15-min session in which we presented 50 different simple and complex sounds (500 ms; Extended Data Fig. 1), new for the animal, each repeated 12 times and delivered in a random order. After sound presentation, another session without auditory stimuli was performed to evaluate the impact of sound presentations on ongoing activity. Then, the same protocol was repeated under anesthesia; 728 ± 180 (474–955) neurons could be imaged simultaneously in cortical layer 2/3 over a field of view of 1 × 1 mm^2^, covering about a quarter of the entire auditory cortex. After automated segmentation of the regions of interest (ROIs) corresponding to the neurons^21^, we estimated the time of putative action potentials based on raw fluorescence signal using the MLSpike deconvolution algorithm^22^ (Extended Data Fig. 1). This yielded population activity rasters for well-identified neurons across sessions and physiological states (Fig. 1c,d and Extended Data Fig. 1), the temporal resolution of which equaled the scan rate of the two-photon microscope (30 Hz).

In line with previous observations^5,23–25^, inspection of the raster plots and the instantaneous population firing curves revealed short synchronous population activity events, which occurred in both the anesthetized and the awake states, but seemed more contrasted and stereotypical under anesthesia compared with the awake state (Fig. 1c,d and Extended Data Fig. 1). We extracted these events by applying a baseline-corrected^26^ threshold to the population firing rate above which a synchronous event could not be explained by the fluctuations of summed independent Poisson’s processes (Fig. 1c,d and Extended Data Fig. 1). Population events were short but of variable duration (327 ± 131 ms, *n* = 11 mice in awake state; 414 ± 237 ms, *n* = 6 mice under anesthesia) and appeared during both the stimulation-free (0.42 ± 0.1 Hz, *n* = 11 mice in awake state; 0.51 ± 0.11 Hz, *n* = 6 mice under anesthesia) and the sound-delivery protocols (Fig. 1c). The percentage of neurons recruited in each population event was affected very little by anesthesia, with only a slight increase in ongoing event size (Fig. 1e). Relying on the same detection criterion, the probability that a population event appeared during sound presentation was 52 ± 20% (*n* = 11 mice) in the awake state and 41 ± 8% (*n* = 6 mice) under anesthesia, with a large disparity across sounds. Some sounds drove detectable population events on almost every trial, but others did not (Fig. 1f). The probability of evoking a population event significantly changed for many sounds between wakefulness and anesthesia (Fig. 1f) in line with previous reports^18,19^, suggesting that anesthesia reorganizes cortical responses.

### Indistinguishable ongoing and evoked events under anesthesia

**Fig. 2:**
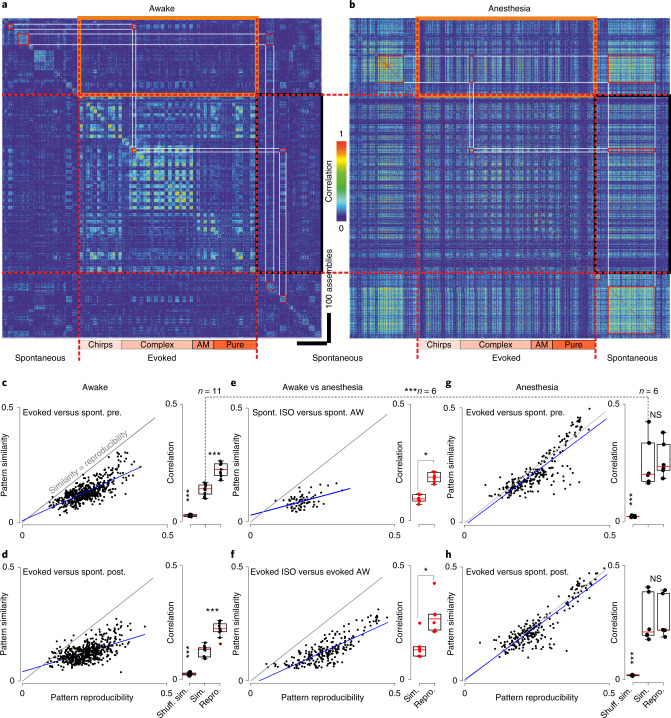
Ongoing assemblies and sound-evoked responses differ in the awake state but overlap under anesthesia. **a**, For an example recording session, Pearson’s correlation matrix between spontaneous assemblies sorted by hierarchical clustering and single trial sound response patterns (whether or not a population event was detected), sorted sound by sound (12 trials per sound, sound order indicated below). Clustering is done independently in pre- and poststimulation periods. Lower correlation inside black and orange frames (similarity) compared with correlations along the diagonal (reproducibility) indicate that spontaneous and evoked patterns are different. **b**, Correlation matrix under anesthesia for the same neuronal population as in **a**. Similar correlation in black and orange frames (similarity) and in the squares along the diagonal (reproducibility) indicates that spontaneous and evoked assemblies are highly similar. **c**–**h**, Relationship between reproducibility (abscissa) and similarity (ordinate) of sound-evoked and spontaneous patterns for all sounds and sessions. Statistics across sessions are given on the right-hand-side boxplots. Spontaneous and evoked patterns can be considered dissimilar if their reproducibility is significantly larger than their similarity (gray shows the line for the equality and dark blue the data trend). A significant difference between spontaneous and evoked patterns is seen in awake mice (*P* = 0.001, Shuffl. Sim. *P* = 0.0002 (**c**); *P* = 0.001, Shuffl. Sim. *P* = 0.0002 (**d**); Wilcoxon’s signed-rank test, n = 11 mice), but not under anesthesia (*P* = 0.44, Shuffl. Sim. *P* = 0.0002 (**g**); *P* = 0.56, Shuffl. Sim. *P* = 0.0002 (**h**); Wilcoxon’s signed-rank test, *n* = 6 mice) where the similarity increased significantly (dashed line, **c** versus **g**, *P* = 0.0003, Wilcoxon’s rank-sum test). Evoked and spontaneous population activity patterns under anesthesia are different from patterns in the awake state (**e**–**f**, *P* = 0.03, *P* = 0.03; paired Wilcoxon’s sgned-rank test, *n* = 6 mice). AM, Amplitude-modulated sounds; NS, not significant; Pure, Pure tones; spont., spontaneous; Repro., reproducibility; Shuffl. sim., similarity for shuffled data; Sim., simulated. ^*^P < 0.05, ^***^P < 0.001. For all box-and-whisker plots, the red mark indicates the median, and the bottom and top edges of the box indicate the 25th and 75th percentiles, respectively. The whiskers extend to the extreme data points. All tests are two sided. ﻿ISO, isoflurane; AW, awake; pre., pre-stimulation. Source data

**Extended Data Fig. 2:**
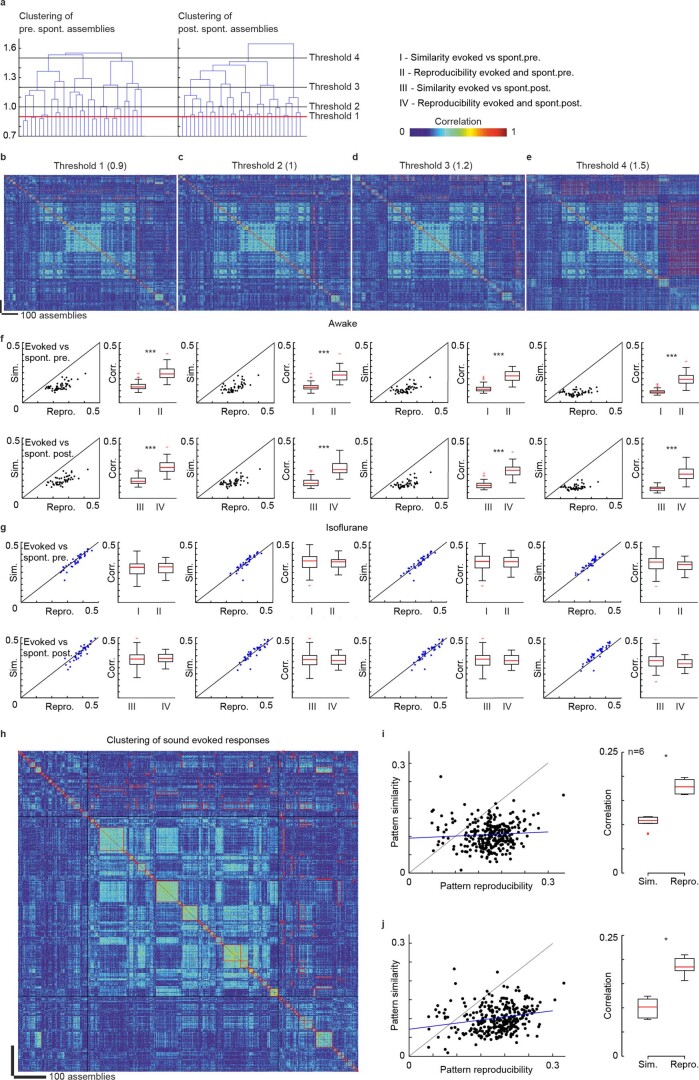
Dissimilarity of evoked and ongoing assemblies is robust to clustering methods and parameters. **a**, Dendrogram plot of the hierarchical binary cluster trees for spontaneous assemblies before and after stimulation in a sample recording session. The horizontal lines indicate the 4 cophenetic distance thresholds tested in the following panels (0.9, 1, 1.2, 1.5). **b-e**, Correlation matrix for a sample recording in the awake state. The spontaneous assemblies are sorted by hierarchical clustering with thresholds 1, 2, 3, and 4 (in **b**, **c**, **d**, and **e** respectively) as defined in **a**. Red squares in the lateral quadrants indicate the maximum similarity pairs. Red squares in the diagonal quadrants indicate clusters and groups. **f**, Scatter plot and statistics of similarity vs reproducibility (top: evoked/spont. pre.; bottom: evoked/spont.post.) for each threshold value for the example shown in **b-e**. Reproducibility of spontaneous and evoked assemblies is significantly higher than the similarity of evoked vs spontaneous assemblies independently of the threshold (all p-values equal to 10^−9^, n = 50 pairs of clusters, Paired Wilcoxon Signed Rank Test). **g**, Same as in **f**, but for the anesthetized state under isoflurane in the same sample population (all p-values were above 0.05, n = 50 pairs of clusters, Paired Wilcoxon Signed Rank Test:thresholds: 0.6 (p = 0.66, 0.50), 0.8 (p = 0.43, 0.94), 1 (p = 0.80, 0.94) and 1.2 (p = 0.16, 0.09)). **h**, Sample matrix where evoked assemblies were clustered in the same way as spontaneous ones (instead of grouping by sound). **i**, Relation between reproducibility (abscissa) and similarity (ordinate) of sound-evoked and spontaneous pre-stimulation assemblies for all sounds and sessions. Statistics across sessions are given in the right-hand-side histograms (p = 0.03, n = 6 mice). **j**, Same as in **i** for evoked *versus* spontaneous assemblies detected post-stimulation (p = 0.03, n = 6 mice). Paired Wilcoxon Signed Rank Test for **i** and **j**. (Corr., Correlation; Repro., Reproducibility; Sim., Similarity; spont, spontaneous; *, *** indicate p < 0.05, p < 0.001 respectively). For all box-and-whiskers plots, the red mark indicates the median, and the bottom and top edges of the box indicate the 25th and 75th percentiles, respectively. The whiskers extend to the most extreme data points not considered outliers, and the outliers are plotted individually using crosses. All tests are two-sided.

**Extended Data Fig. 3:**
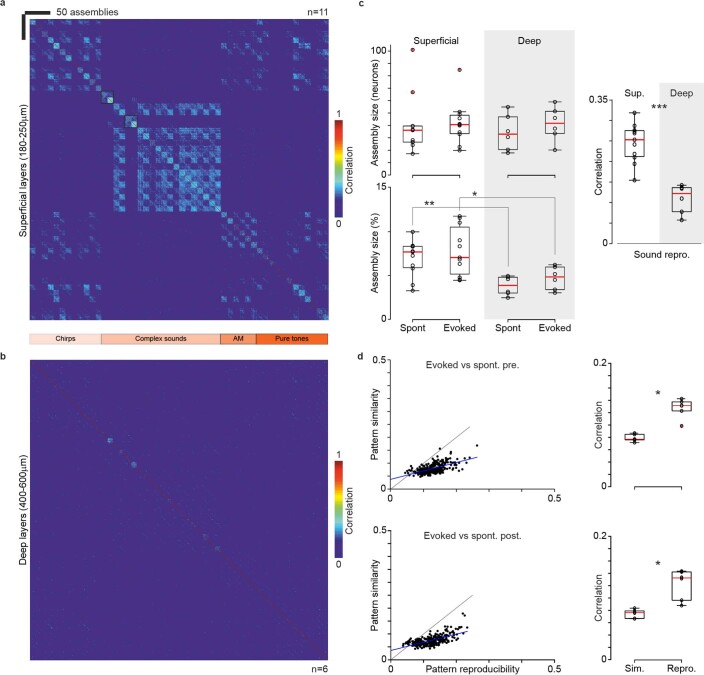
Dissimilarity of evoked and ongoing assemblies is seen also in deep cortical layers. **a**, Pearsons’s correlation matrix averaged across 11 mice for the assemblies evoked by sounds in the upper layers of auditory cortex (180–250 um below cortical surface). **b**, The same as in **a**, in deep layers (450–600 um below the cortical surface) averaged across 6 mice. **c**, Top left: average size (number of neurons) of neuronal assemblies in superficial (11 mice) and deep layers (6 mice). Bottom left: average size of neuronal assemblies in % of all neurons in the FOV in superficial and deep layers (p = 0.005, p = 0.02, Wilcoxon rank sum test). Right: sound evoked responses reproducibility in superficial layers is higher than in the infragranular layers (p = 0.0002, Wilcoxon rank sum test). For all tests in c, n = 11 mice for superficial, and n = 6 mice for deep. **d**, Dissimilarity of sound-evoked and spontaneous patterns for all sounds and sessions in deep layers. Statistics across sessions are given on the right-hand-side histograms (p = 0.03, p = 0.03, Paired Wilcoxon Signed Rank Test, n = 6 mice). (Repro., Reproducibility; Sim., Similarity; Spont, spontaneous; Sup., Superficial; *,**,*** indicate p < 0.05, p < 0.01, p < 0.001 respectively). For all box-and-whiskers plots, the red mark indicates the median, and the bottom and top edges of the box indicate the 25th and 75th percentiles, respectively. The whiskers extend to the most extreme data points. All tests are two-sided.

**Extended Data Fig. 4:**
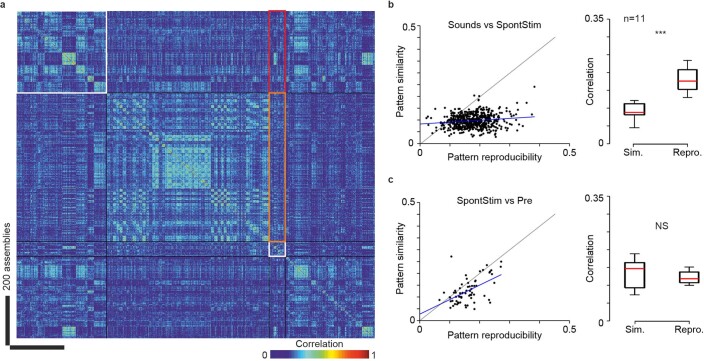
Ongoing assemblies during stimulation are also dissimilar to evoked responses. **a**, For an example recording session, Pearson’s correlation matrix between spontaneous assemblies sorted by hierarchical clustering and single trial sound response patterns. Spontaneous assemblies detected during stimulation sessions in between evoked responses are clustered in the small white rectangle. Their similarity with evoked and spontaneous pre-stimulation assemblies (big white rectangle) are within orange and red rectangles, respectively. **b**, Dissimilarity of sound-evoked and spontaneous patterns (SpontStim) detected during stimulation (reproducibility on abscissa and similarity on ordinate). Statistics across sessions are given on the right-hand-side histograms (p = 0.001). **c**, Similarity of ongoing assemblies observed during the pre-stimulation and stimulation epochs. (p = 0.41, Paired Wilcoxon Signed Rank Test, n = 11 mice in **b** and **c**). (NS, not significant; Repro., Reproducibility; Sim., Similarity; Spont, spontaneous; *** indicates p < 0.001). For all box-and-whiskers plots, the red mark indicates the median, and the bottom and top edges of the box indicate the 25th and 75th percentiles, respectively. The whiskers extend to the most extreme data points. All tests are two-sided.

To investigate more precisely this reorganization, we then asked whether events observed in the absence of stimuli resemble the activity generated by sensory stimuli, and in particular whether similar assemblies of neurons are recruited in both cases. For ongoing activity, we identified each detected population event to the assembly of neurons that fired at least one putative action potential during the event. To avoid unnecessary thresholding effects, for sound-evoked activity, the assembly of responsive neurons was the neurons that fired at least one putative action potential during sound presentation (response time window: 0–500 ms after sound onset). Similarity between assemblies was measured based on the correlation between binary population vectors (entry 1 for neurons belonging to the assembly and 0 otherwise), which had the length of the entire neuronal population. Then we used a simple approach to account for the intrinsic variability of neural responses and noise introduced by spike estimation errors^22^. We performed hierarchical clustering to organize all ongoing assemblies in groups of similar patterns (independently for ongoing activity before and after stimulation) and displayed the matrix of pairwise similarity between individual ongoing assemblies and single trial responses to all tested sounds (Extended Data Fig. 1 and Fig. 2a,b). Visual inspection of sample matrices for pairs of sounds and ongoing assembly clusters indicated, overall, a low similarity between ongoing assemblies and evoked sound responses in the awake state, but a high similarity between ongoing assemblies and evoked sound responses in the same neuronal population under isoflurane anesthesia (Fig. 2a,b). To quantify this, we measured, for each sound and imaging session, the mean similarity between individual evoked responses and the assemblies of the most similar ongoing assembly cluster. To evaluate to what extent similarity is limited by the variability of responses or spontaneous patterns, we defined the reproducibility of evoked responses as the mean population activity correlation across repetitions of the same stimulus. Reproducibility of spontaneous clusters was defined as the mean correlation between the assemblies within a cluster. Plotting similarity against the mean of spontaneous and evoked assembly reproducibility, it became evident that similarity was below reproducibility levels in the awake state whether ongoing assemblies were taken before (Fig. 2c) or after (Fig. 2d) sound presentations. This result was robust to changes in clustering parameters (Extended Data Fig. 2) and cortical depth (Extended Data Fig. 3). It also held for assemblies observed in between sound stimulations (Extended Data Fig. 4) and when ongoing assemblies were compared with sound responses clustered in the same way (Extended Data Fig. 2). Hence, evoked responses and ongoing assemblies correspond to distinct population activity patterns in awake, freely listening mice.

**Extended Data Fig. 5:**
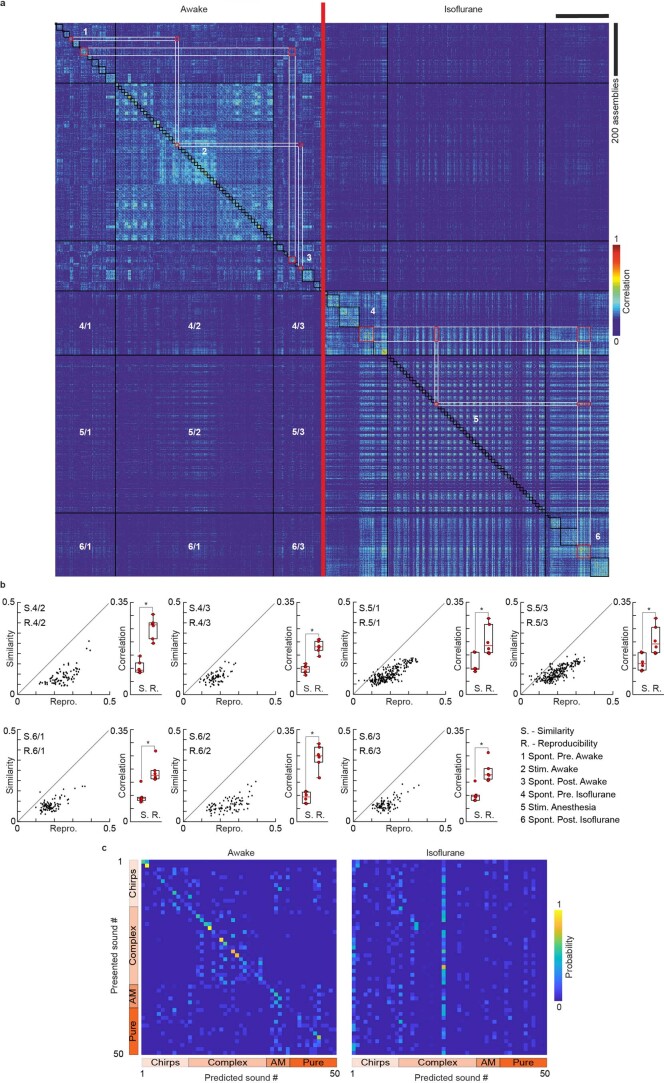
Transition from awake to anesthesia changes both sound-evoked and spontaneous patterns. **a**, Correlation matrices for ongoing and evoked assemblies across wakefulness and anesthesia. The numbering of the quadrant describes the different recording conditions. (1) Ongoing assemblies in the awake state, sorted by hierarchical clustering (black rectangles). (2) Single trial sound responses in the awake state, grouped sound-by-sound (black rectangles). (3) Post-stimulation ongoing assemblies in the awake state. (4) Ongoing assemblies under anesthesia, before stimulation. (5) Single trial sound responses under anesthesia (whether or not a population event was detected). (6) Ongoing assemblies under anesthesia, after stimulation. Red rectangles in the top right quadrant outline the similarity between the assemblies detected in the awake and anesthetized states. Numbering in the bottom left quadrant indicates pairwise comparisons (for example 4/1 Spont. Pre. Anesthetized vs Spont. Pre. Awake). **b**, Plot of awake versus anesthetized assembly similarity against assembly reproducibility. Statistics across sessions are given on the right-hand-side histograms (p = 0.03, Paired Wilcoxon Signed Rank Test, n = 6 mice). **c**, Confusion matrix (that is probability) of the classifier used in Fig. 4e averaged across the 5 mice in which the same populations were imaged in the awake and isoflurane anesthetized state. (AM, amplitude modulated sounds; Repro., Reproducibility; Pure, Pure tones; * indicates p < 0.05). For all box-and-whiskers plots, the red mark indicates the median, and the bottom and top edges of the box indicate the 25th and 75th percentiles, respectively. The whiskers extend to the most extreme data points not considered outliers, and the outliers are plotted individually using red crosses. All tests are two-sided.

However, under isoflurane anesthesia, a profound reorganization of the cortical dynamics was observed. First, ongoing assemblies and sound responses seen under anesthesia were clearly distinct from those seen in the awake state (Fig. 2e,f and Extended Data Fig. 5). Second, evoked responses and ongoing assemblies were highly similar under isoflurane anesthesia, as measured through the equal similarity and reproducibility levels of particular sound responses and with at least one cluster of spontaneous assemblies (Fig. 2g,h and Extended Data Fig. 5).

**Fig. 3:**
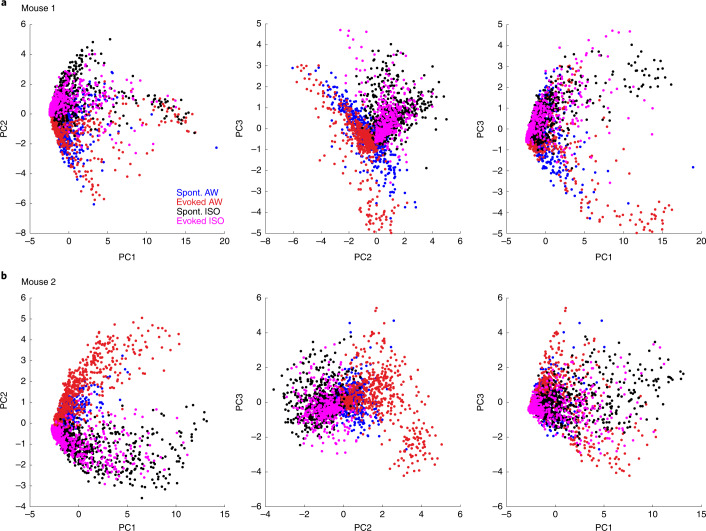
Assemblies of the awake and anesthetized states span different regions of the population state space. **a**, Plot of the localization of ongoing and evoked assemblies in the state in the 3D space defined by the first three PCs of the dataset including all ongoing and evoked assemblies, for one representative recording sample. Anesthesia is with isoflurane. Three 2D projections are shown, from left to right: planes defined by 1st and 2nd PCs, by 2nd and 3rd PCs and by 1st and 3rd PCs. **b**, Same as **a** for another sample recording in a different animal. Anesthesia is with isoflurane. Color code: magenta = evoked isoflurane (Evoked ISO), red = evoked awake (Evoked AW), black = spontaneous isoflurane (Spont. ISO), blue = spontaneous awake (Spont. AW).

Overall, the massive transformation of assemblies from wakefulness to anesthesia can also be qualitatively summarized by plotting the localization of ongoing assemblies in the neuronal state space after dimensionality reduction (that is, in the space of the first three principal components (PCs) of a dataset including all assemblies and responses; Fig. 3). In this format, it becomes evident that assemblies observed in the awake state are essentially distinct from assemblies observed under isoflurane anesthesia. Moreover, under anesthesia, sound responses and ongoing assemblies span the same region of the neuronal state space, whereas in wakefulness they clearly span different regions (Fig. 3). In the awake state, spontaneous and evoked activity can thus be easily distinguished, whereas under isoflurane anesthesia, a sound response leads to population activity patterns that are extremely similar to ongoing activity. Therefore, sound responses under isoflurane are much harder to interpret as a stimulus to be perceived in downstream targets of the auditory cortex, which could potentially contribute to the lack of perceptual reports despite the existence of cortical responses.

**Extended Data Fig. 6:**
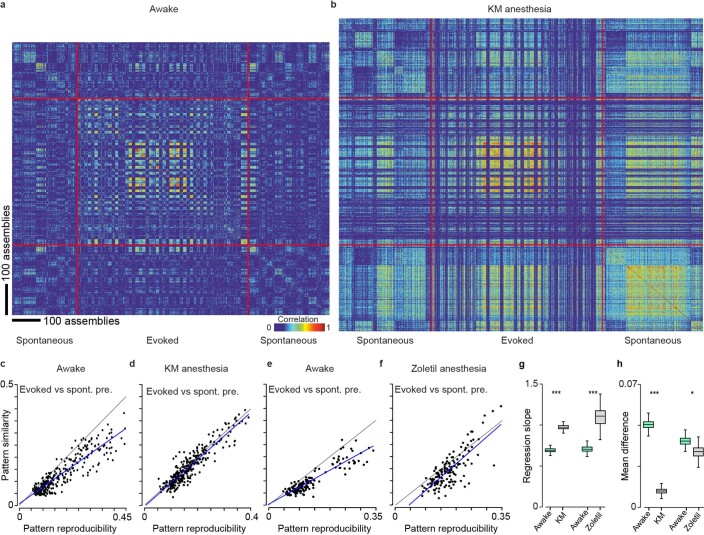
Ongoing assemblies and sound-evoked responses overlap under ketamine-medetomidine and zolazepam-tiletamine anesthesia. **a**, For an example recording session, Pearson’s correlation matrix between spontaneous assemblies sorted by hierarchical clustering and single trial sound response patterns (whether or not a population event was detected), sorted sound by sound (12 trials/sound). Clustering is done independently in pre- and post-stimulation periods. Lower correlation inside black and orange frames (similarity) compared to correlations along the diagonal (reproducibility) indicate that spontaneous and evoked patterns are different. **b**, Correlation matrix under ketamine (50 mg/kg) medetomidine (1 mg/kg) anesthesia (KM) for the same neuronal population as in **a**. Similar correlation in black and orange frames (similarity) and in the squares along the diagonal (reproducibility) indicate that spontaneous and evoked assemblies are highly similar. **c**, Relation between reproducibility (abscissa) and similarity (ordinate) of sound-evoked and spontaneous patterns for the 50 sounds and 6 sessions recorded in 3 mice. The unity line in black. Regression line in blue. **d**, Same as in **c**, but under KM anesthesia. **e-f**, Same as in **c, d**, but for 3 experiments in 3 different mice for Zoletil® (70 mg/kg) anesthesia. **g**, Slope of the regression lines in **c**-**f**. Box plot indicates the 5% confidence interval obtained by bootstrapping 1000 times across data points. For both anesthetics, the bootstrap p-value was <0.001. **h**, Mean difference between reproducibility and similarity in **c**-**f**. The bootstrap p-value was <0.001 for KM and 0.02 for Zoletil. In boxplots of **g-h**, the central mark indicates the median, and the bottom and top edges of the box indicate the 25th and 75th percentiles, respectively. Distributions were obtained by bootstrapping across data points. The whiskers extend to the most extreme data points. Bootstrapping (1000 random resampling with replacement) was also used to assess the significance of the difference between slopes obtained in anesthesia and in wakefulness. For both anesthetics, the bootstrap p-value was <0.001. For KM anesthesia, the difference between awake and anesthetized was also significant when testing across the 6 sessions (Wilcoxon rank sum test, p = 0.03). Note that the higher slopes observed here in the awake state compared to the results shown in Fig. 2 are likely due to the smaller field of views used in KM and Zoletil® experiments (see Methods), which reduced the variety of assembly configurations and therefore the distance between assemblies. Note also that, in Zoletil anesthesia, reproducibility of sound responses was higher on average than their similarity with spontaneous activity; however, this effect was restricted to less reproducible sound response patterns, and most reproducible sound responses were similar to spontaneous activity as seen in isoflurane and KM anesthesia. This is in contrast with the awake state in which most reproducible sound responses are clearly distinct from spontaneous activity. The clear change between awake and Zoletil® anesthesia for most reliable responses is better captured by the slope measurements in **g**. (KM, ketamine medetomidine; spont. pre., spontaneous pre-stimulation; *,*** indicate p < 0.05, p < 0.001 respectively). All tests are two-sided. Source data

**Extended Data Fig. 7:**
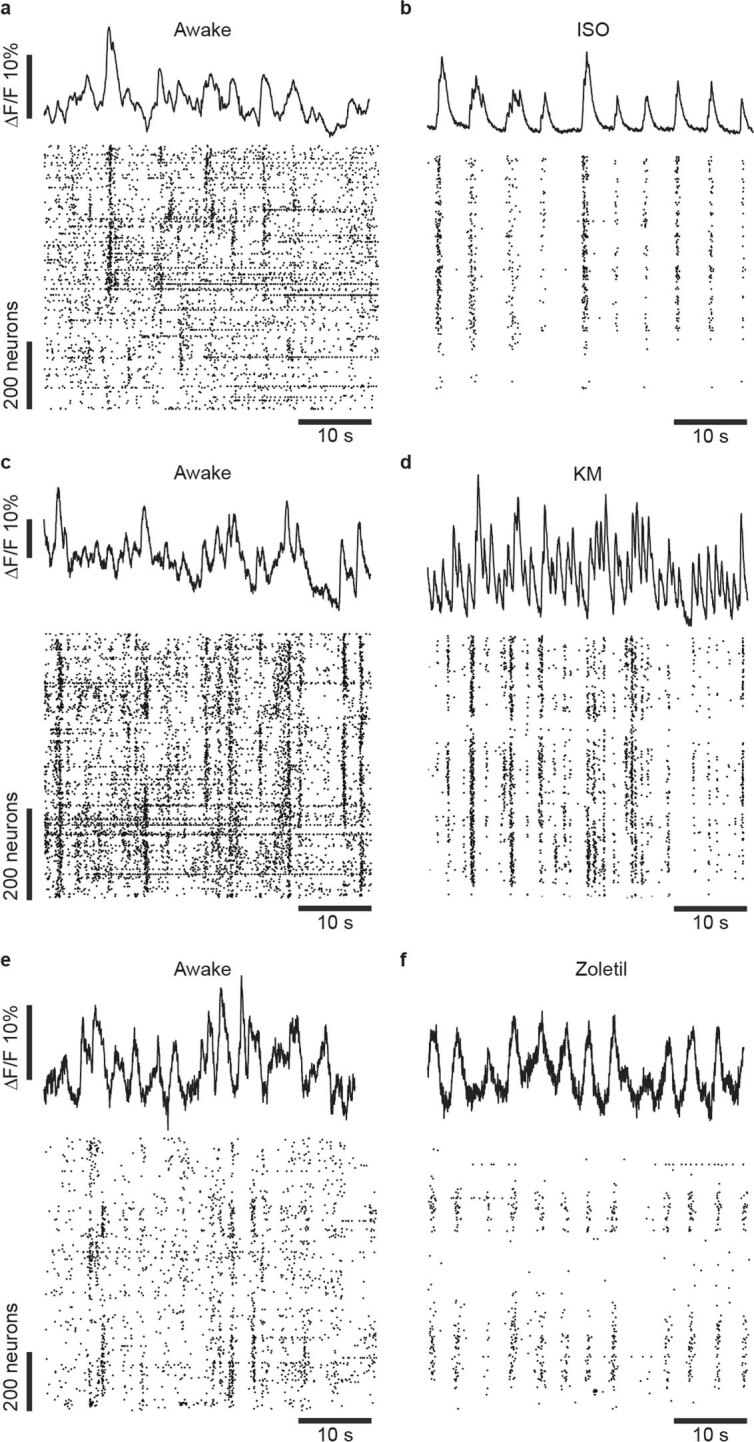
Sample neuropil traces during wakefulness and anesthesia. **a**, Top panel: Sample trace of the mean neuropil signal for a representative imaging session during the awake state. Neuropil was measured in a disk of 20 pixels radius around each region of interest identified as neurons, excluding other neurons. Bottom panel: Raster plot of putative action potentials (MLSpike) in the recorded population align with the neuropil trace above. **b**, Same as in **a**, but under isoflurane anesthesia (ISO). **c**, **d**, Same as in **a, b**, but for ketamine medetomidine anesthesia (KM). **e**, **f**, Same as in **a**, **b**, but for Zoletil anesthesia.

To further probe this idea, we repeated the experiment with other anesthetics that have different modes of action. Following the same neuronal populations in the auditory cortex before and after anesthesia induced by subcutaneous injection of the classic mix of 50 mg kg^−1^ of ketamine and 1 mg kg^−1^ of medetomidine (KM), we observed that evoked responses and ongoing assemblies were extremely similar under KM anesthesia, unlike in the awake state (Extended Data Fig. 6). This is remarkable because KM, unlike isoflurane, is a dissociative anesthetic that targets *N*-methyl-d-aspartate receptors. In the mouse, KM also leads to faster cortical rhythms than isoflurane because we could measure for the neuropil signal, a proxy of echocardiography signals^27^ (Extended Data Fig. 7). Often used in auditory neurophysiology experiments, KM provides long-lasting anesthesia in mice and can produce deep anesthesia. We therefore also repeated our experiments with the lighter dissociative anesthetic Zoletil, a 50:50 mix of tiletamine and zolazepam (70 mg kg^−1^) that maintains cardiorespiratory function at a high level. We again observed similar ongoing and evoked assemblies under anesthesia (Extended Data Fig. 6), in particular for most reproducible assemblies, despite shorter anesthesia duration (typically ~40 min) and a different associated brain rhythm (Extended Data Fig. 7). Note that we could verify, for all three anesthetics, that population activity followed global up and down states, which is a clear sign of anesthesia (Extended Data Fig. 7).

### Impoverished cortical sound representations under anesthesia

**Fig. 4:**
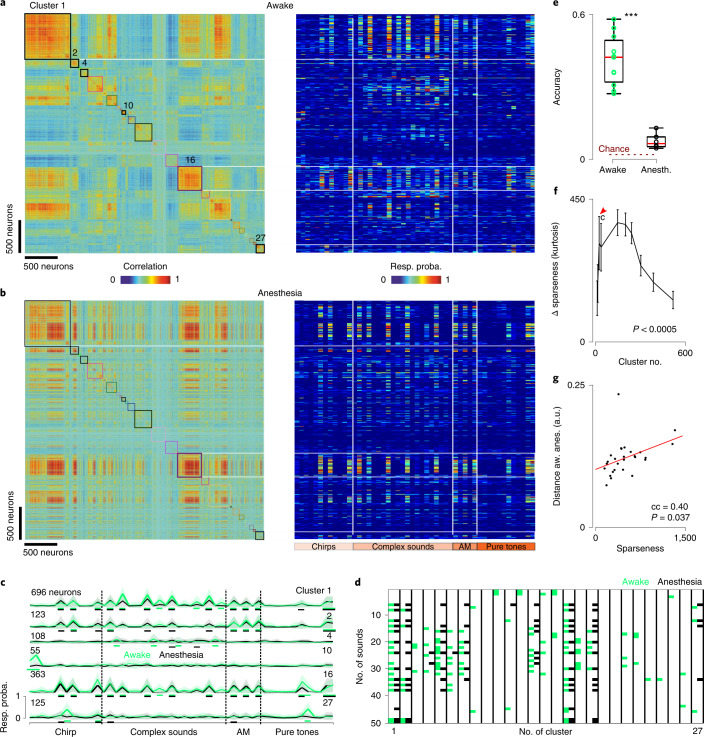
Modification of sound tuning between wakefulness and anesthesia. **a**, Left: matrix of response profile correlation for 3,641 neurons in six awake mice. The neurons are clustered according to the similarity of their responses (metric: Pearson’s correlation coefficient between response probability profiles for the 50 sounds). Right: response probability (Resp. proba.) profiles for all recorded neurons organized with the clustering presented to the left. **b**, Same as **a** under anesthesia. **c**, Mean response profiles in the awake state (green) and under anesthesia (black) for the six sample clusters labeled in **a**. Green and black rectangles represent significant responses; error bands represent the s.d. around the mean. **d**, Colored rectangles denote sounds producing a significant response in neurons of 27 clusters presented in **a**. A multicomparison one-way ANOVA between the actual responses and a shuffled surrogate of the responses was used for the significance evaluation. **e**, Average sound prediction accuracy for the 50 sounds in the awake versus the anesthetized (Anesth.) state (*n* = 11 and 6, *P* = 0.0002, Wilcoxon’s rank-sum test, ^***^P < 0.001). Box-and-whisker plots show the red mark denoting the median, and the bottom and top edges of the box indicating the 25th and 75th percentiles, respectively. The whiskers extend to the extreme data points. **f**, Difference of sparseness of cluster responses to sounds (that is, kurtosis of response distribution) between awake and anesthetized states, as a function of the granularity number of the clustering algorithm. Positive values indicate higher sparseness in the awake state. All measures are significantly larger than zero (Wilcoxon’s signed-rank test; all *P* values are <0.0005; *n* is the number of clusters given on the *x* axis, mean values ± s.e.m.). **g**, Difference between sound responses in the awake state and under anesthesia (Distance aw. anes.) as a function of response sparseness for 27 clusters selected in **a** (*P* = 0.037, bootstrap test, no multiple comparison adjustment). a.u., arbitrary units; cc, correlation coefficient. All tests are two sided. Source data

These results also suggest that assemblies in ongoing activity and sensory responses emerge from a more stereotypical process in anesthesia, whereas in the awake state the auditory cortex develops a richer repertoire of activity patterns that carry more information, in particular about sounds. To investigate this, we reordered all neurons imaged in both conditions based on a hierarchical clustering of their trial-averaged response signatures to our 50 sounds in the awake state (Fig. 4a). The number of clusters was chosen to be close to the maximum dimensionality of the pool of response signatures, which is limited by the number of sounds^28^. This revealed a variety of response signatures that corresponded to groups of neurons of different sizes, as plotted in the heatmap of Fig. 4a. We then plotted the heatmap corresponding to sound response signatures during isoflurane anesthesia with the same order of the neurons (Fig. 4b). This revealed that many of the neurons displaying responses that were specific to a few sounds in the awake state responded under anesthesia with either an absence of responses or a less specific and more stereotypical response signature (Fig. 4b). This was also clearly visible when plotting the mean response signatures for awake and anesthetized states for a few representative clusters (Fig. 4c). Several clusters showed highly significant changes in their responses. For a few of them (for example, clusters 1 and 16), this corresponded to minor changes in response magnitudes. However, for many clusters, isoflurane anesthesia drastically changed their mean response signature, often due to the disappearance of some sparse, specific responses (Fig. 4d). This suggests that the sound representation was strongly impoverished by isoflurane anesthesia. It is interesting that the few clusters with response signatures that were preserved under anesthesia (representing 43% of neurons) suggest the existence of more robust response modes (for example, Fig. 4a–c, clusters 1, 2 and 16).

To quantify the impoverishment of sound-evoked assembly patterns, we evaluated the information carried by sound-evoked population responses in each imaging session, using a crossvalidated template-matching classification algorithm. Corroborating our qualitative observations, overall sound decoding performance drastically dropped under anesthesia (41 ± 10% in awake; 7 ± 3% in anesthesia), without reaching the 2% chance level (Fig. 4e). Moreover, the structure of prediction errors was strongly modified between the awake state and the anesthetized state (Extended Data Fig. 5), corroborating the profound change of sound representations at a population scale under anesthesia.

One reason for the lower sound information despite continued sound responses is that responses of individual auditory cortex neurons tended to be less specific to particular sounds under isoflurane anesthesia (Fig. 4d). This could be quantified with a generic lifetime sparseness measure, the kurtosis of the response distribution^29^, across a wide range of clustering sizes (Fig. 4f). Moreover, sparser clusters were more affected by anesthesia (Fig. 4g), indicating that, even if anesthesia spares a large fraction of sound responses, it tends to abolish the most specific ones. Therefore, isoflurane anesthesia produced a strong impoverishment of sound representations, which preserved only a small fraction of the sound information that was present in the same cortical neuron population in the awake state.

### Distinct spontaneous and evoked events in thalamic axons

**Fig. 5:**
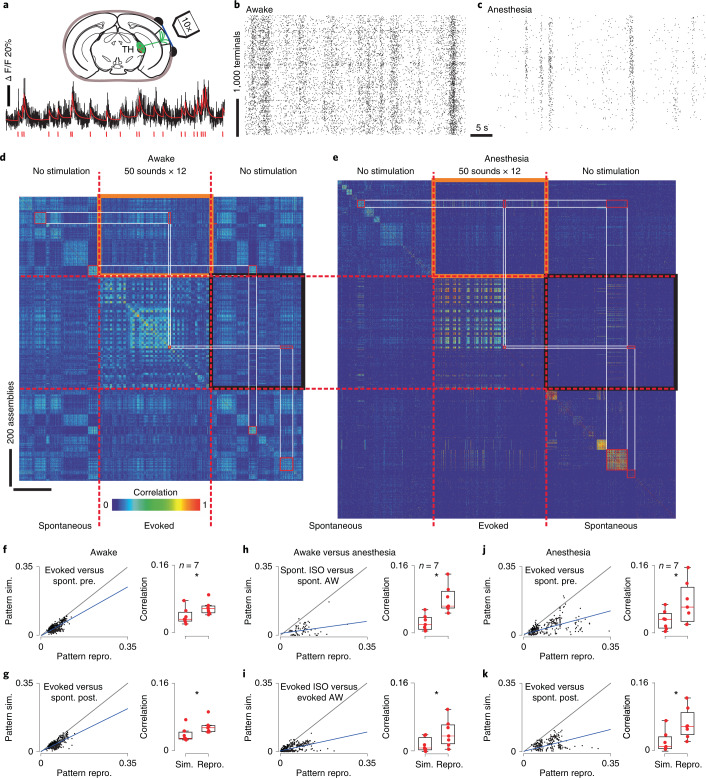
Ongoing and evoked population activity patterns in auditory thalamus differ in both awake and anesthetized states. **a**, Schematic of the procedure for two-photon Ca^2+^ imaging of thalamocortical terminals expressing GCaMP6s in layer 1 of the auditory cortex. Below: calcium trace (black) with spike time estimates (red) for a sample terminal. **b**, Population raster plot in the awake state (each line represents the spontaneous spiking pattern of a thalamocortical terminal). **c**, Same as **b** under light isoflurane anesthesia (1.3%). **d**, For an example recording session, Pearson’s correlation matrix between spontaneous assemblies of thalamocortical terminals sorted by hierarchical clustering and single trial sound response patterns (whether or not a population event was detected), sorted sound by sound (12 trials per sound). **e**, Same as **d** under anesthesia. Lower correlation inside black and orange frames (similarity) compared with correlations along the diagonal (reproducibility) indicates that spontaneous and evoked patterns are different. **f**–**k**, Relationship between reproducibility (abscissa) and similarity (ordinate) of sound-evoked and spontaneous patterns for all sounds and sessions. Statistics across sessions are given on the histograms on the right-hand side (**f**–**k**: *P* = 0.016, paired Wilcoxon’s sgned-rank test, *n* = 7 mice). Spontaneous and evoked patterns are dissimilar in both awake (**f** and **g**) and anesthetized (**j** and **k**) states (gray, line of equality; dark blue, data trend) and neither of them kept similitude passing from one state to another (**h**–**j**). AW, awake; ISO, isoflurane anesthesia; post., poststimulation; pre., before stimulation; repro., reproducibility; Sim., similarity; spont., spontaneous; TH, thalamus. ^*^P < 0.05. For all box-and-whisker plots, the red mark indicates the median, and the bottom and top edges of the box indicate the 25th and 75th percentiles, respectively. The whiskers extend to the extreme data points. All tests are two sided. Source data

To evaluate the contribution of thalamic inputs in the reorganization of cortical activity, we imaged thalamic boutons in the auditory cortex labeled with GCaMP6s through stereotaxic adeno-associated virus (AAV) injections in the primary and secondary auditory thalamus (Fig. 5a)^30,31^. Signals from individual thalamic axons and boutons were extracted using the same automated methods as for somatic fluorescence, applied to a smaller field of view in layer 1 of the auditory cortex. Using the MLSpike algorithm, we obtained estimates of the spike trains (Fig. 5a) underlying thalamocortical synaptic release in the cortex. As in the cortex, population activity in the thalamus displayed short synchronous population events, which more clearly departed from baseline population activity under anesthesia (Fig. 5b,c). Measuring population vector correlation as previously (Fig. 5d,e), we observed that ongoing assemblies were significantly different from evoked activity in the awake state in the thalamic output (Fig. 5f,g), as we had also observed in the cortex. Thus, the thalamic input provides separate information streams, based on which the cortex can build distinct assemblies in its ongoing and evoked activity.

**Extended Data Fig. 8:**
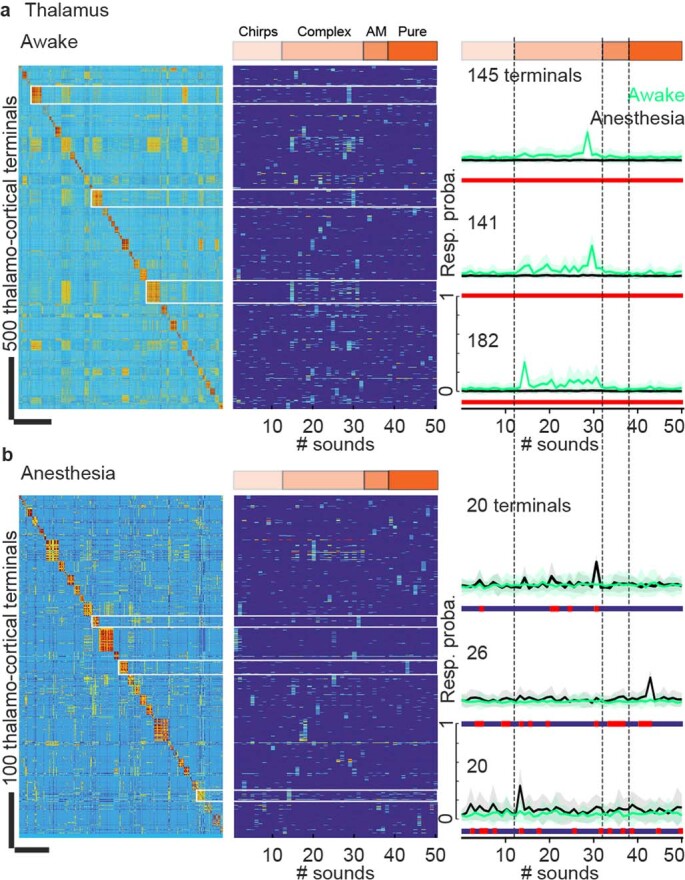
Transformation of thalamic sound tuning profiles between wakefulness and anesthesia. **a**, Left panel: a fragment of response profile correlation matrix containing 2800 of 13314 clustered responsive thalamo-cortical terminals (7 mice) recorded in the layer 1 of the auditory cortex in the awake state. Middle panel: Response probability for thalamo-cortical terminals organized with the same clustering as for the matrix of the left panel (colors in the upper band indicate the type of sound). Right panel: Mean response profiles of thalamo-cortical terminals in the awake state (green trace) and under anesthesia (black trace) for the 3 sample clusters labeled in the left panel. Error bands indicate standard deviation around the mean. As for the cortical neurons, tuning profiles of thalamo-cortical fibers are significantly modified (red rectangles underneath the traces indicate a significant difference; two-sided Wilcoxon rank sum test, using a p-value threshold of 0.05) when changing from the awake to anesthetized state. (AM, Amplitude modulated sounds; Pure, Pure tones; Resp. proba., Response probability). Error bands indicate standard deviation around the mean. b, Same as a for another fragment of the same correlation matrix, now containing 620 of 13314 responsive thalamo-cortical terminals.

It is interesting that, under isoflurane anesthesia, neural assemblies in the thalamic input were also profoundly reshaped compared with assemblies seen in the awake state (Fig. 5h,i). This involved changes in the tuning of individual neurons to sounds, as we had also observed in the auditory cortex (Extended Data Fig. 8). During anesthesia, but not during wakefulness, thalamic activity patterns were usually different across our two spontaneous activity-recording sessions (before versus after sound stimulation; Fig. 5e). This may reflect the fluctuations in the depth of anesthesia typically observed in such narcosis conditions. However, unlike what we observed in cortical neurons, ongoing assemblies observed before or after sound presentation in thalamic fiber activity were clearly different from evoked activity patterns, as seen from reproducibility levels that were at least 2× larger than similarity levels (Fig. 5j,k). Therefore, during isoflurane anesthesia, the thalamus sends sensory-driven and ongoing activity inputs that are different, but cortical activity does not take this difference into account.

### Specific cell populations for evoked and spontaneous events

**Fig. 6:**
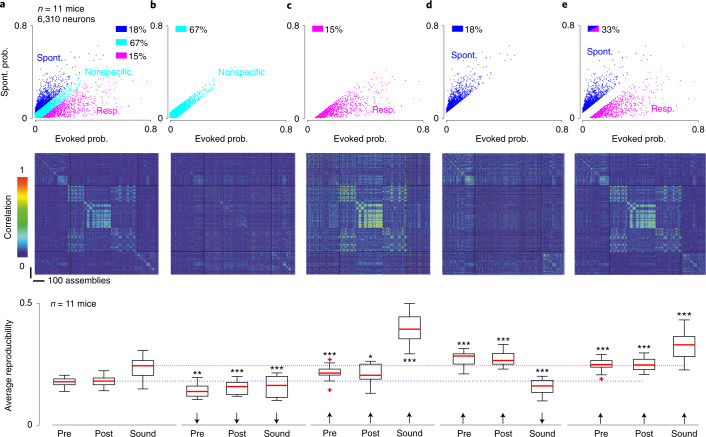
Specificity of evoked patterns relies on a subset of neurons in the awake state. **a**, Top: plot of the probability of responding to any sound versus the probability of being recruited in an ongoing event for 6,310 neurons in 11 mice. The color code indicates whether the neuron prefers ongoing (blue) or evoked (magenta) events or nonspecifically participates in both types of events (cyan, boundaries: ±1 m.a.d. of the probability difference). Middle: for a sample session, Pearson’s correlation matrix was computed with all available neurons. Bottom: average sound and spontaneous event cluster reproducibility. **b**, Same as **a** but correlation matrix and reproducibility (mean correlation across assemblies of the same spontaneous cluster or of the same sound) are calculated with nonspecific neurons only (67%). **c**, Same as **a** but for sound responsive neurons only (15%). **d**, Same as **a** but only for neurons preferring ongoing events (18%). **e**, Same as **a** but for all except nonspecific neurons (33%). Post, poststimulation; Pre, before stimulation; prob., probability; Resp, responsive; Spont, spontaneous. ^*^P < 0.05, ^**^P < 0.01, ^***^P < 0.001 respectively. For box-and-whisker plots, the red mark indicates the median, and the bottom and top edges of the box indicate the 25th and 75th percentiles, respectively. The whiskers indicate the most extreme non-outlier data points. Red crosses are outliers. **b**–**e**: *P* = 0.002/0.001/0.001; *P* = 0.001/0.02/0.001, *P* = 0.001/0.001/0.001, *P* = 0.001/0.001/0.001; paired Wilcoxon’s signed-rank test (*n* = 11 mice). All tests are two sided. Source data

**Extended Data Fig. 9:**
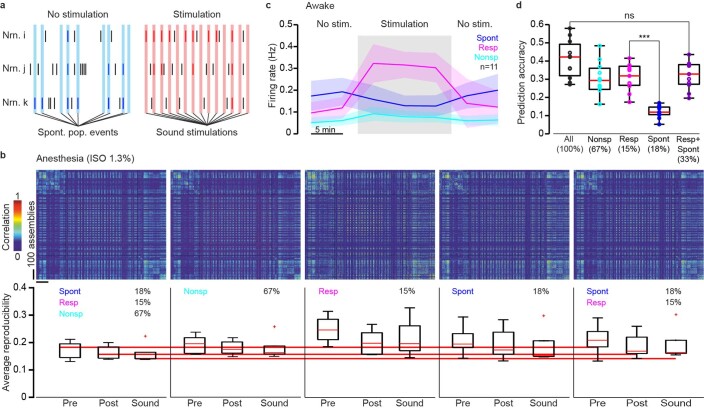
Neuronal assemblies lose their functional specificity under anesthesia. **a**, Schematics depicting the rationale for the selection of the three functional populations defined in Fig. 7. For each neuron the probability to participate in a spontaneous event (blue bars) and in a sound evoked event (red bars) is calculated. **b**, Top left: For a sample session under anesthesia, Pearson’s correlation matrix computed with all available neurons. Bottom left: Average sound and spontaneous event cluster reproducibility under anesthesia. The neurons are divided into 3 groups based on their activity in the awake state before anesthesia application. From left to the right: Same as the first matrix, but only for Nonspecific neurons; only for predominantly sound responsive neurons (Resp); only predominantly spontaneously active neurons (Spont); only for Resp+Spont. Differences between the first panel and subsequent ones were not significant, (Wilcoxon rank sum test, n = 5 mice, p = 0.063 for all differences). **c**, Time course of the mean firing rate profiles for each of the 3 groups of neurons in all awake mice (n = 11). Note that the graph presented in Fig. 7b corresponds to only 5 mice that were recorded both in awake and anesthetized conditions. Error bands indicate standard deviation around the mean. **d**, Average prediction accuracy for all sounds for the 4 sub-populations (n = 11 mice, p = 0.11, p = 0.00001, One-way ANOVA test). (ISO, isoflurane; Nonsp, non-specific; ns, not significant; pop., population; Post, post stimulation; Pre, prior to stimulation; Resp, responsive; Spont, spontaneous; stim., stimulation; *** indicates p < 0.001). For all box-and-whiskers plots, the red mark indicates the median, and the bottom and top edges of the box indicate the 25th and 75th percentiles, respectively. The whiskers extend to the most extreme data points not considered outliers, and the outliers are plotted individually using red crosses. All tests are two-sided.

To find further evidence for this observation, we analyzed single-neuron properties in cortical activity, seeking to identify distinctive functional markers of ongoing and evoked activity. We measured, for each neuron, the probability of emitting at least one putative action potential during a spontaneous assembly and during presentation of a sound (500 ms after onset). In the awake state, the scattering of probabilities was broad (Fig. 6a). Substantial fractions of neurons were more specific to ongoing assemblies (~1/6) or more specific to sound responses (~1/6; Fig. 6a). The remaining neurons had similar probabilities of participating in ongoing assemblies or evoked responses (~2/3; Fig. 6a). Specific neurons were large contributors to the identity and robustness of ongoing assemblies and sound responses. Indeed, if specific neurons were removed by retaining neurons for which the absolute difference between ongoing and evoked probabilities was <1 m.a.d. (mean absolute deviation) of the full distribution, population vector reproducibility levels drastically dropped for both ongoing assemblies and sound responses (Fig. 6b), although some information about sounds remained (Extended Data Fig. 9). Corroborating this, when nonspecific neurons were discarded, population vector reproducibility levels were boosted (Fig. 6c–e). Also, if only spontaneous assembly neurons were retained, reproducible and sound-specific sound response patterns disappeared (Fig. 6d), leading to a severe drop in sound decoding (Extended Data Fig. 9). This indicates that, even if no neuron is fully specific for ongoing or evoked activity, as previously observed in the visual cortex^10^, small and distinct neuronal subpopulations are specialized in carrying sound-specific information versus forming different ongoing activity motifs.

**Fig. 7:**
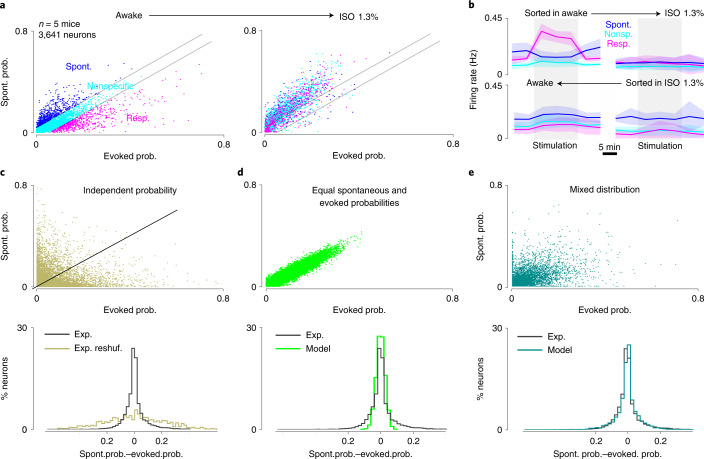
Specificity of cortical neurons for evoked or ongoing activity is redistributed in anesthesia. **a**, Plot of the probability of being recruited in an ongoing event (spont. prob.) plotted against the probability of responding to any sound (evoked prob.) for 3,641 neurons in 5 mice in the awake state (left) and under anesthesia (right). The color code is defined as in the left panel. Under anesthesia, the three color-coded populations converge to a single group with strongly correlated probability of activation in spontaneous and evoked events. **b**, Top: time course of the mean firing rate profiles in the awake state (left) and under anesthesia (right) for each of the three groups of neurons defined in **a** for the awake state. Bottom: same as above but when the responsive, spontaneous and nonspecific groups are defined in anesthesia. Error bands indicate the s.d. around the mean. **c**, Top: same as **a** but probabilities reshuffled along each axis to show the expected probability distribution for independent participations in ongoing and evoked events. Bottom: corresponding distribution of probabilities difference (ongoing − evoked, 6,310 neurons, in 11 mice). Experimental is black and reshuffled dark khaki. **d**, Probability distributions for 6,310 surrogate neurons with equal participation probabilities in ongoing and evoked events. **e**, Probability distributions for 6,310 surrogate neurons with probabilities of participating in spontaneous and evoked activity correlated but not equal (that is, the difference between spontaneous and evoked probabilities is drawn from a continuous Gaussian distribution). Exp., experimental; ISO, isoflurane anesthesia; Nonsp., nonspecific; reshuf., reshuffled; Resp., responsive; Spont., spontaneous.

Tracking nonspecific, sound-specific and ongoing assembly-specific subpopulations during anesthesia further uncovered the profound transformation of cortical dynamics. Plotting the probability of firing in an ongoing assembly against the probability of firing in a sound-evoked response indicated that the two specific neuronal subpopulations were strongly redistributed under isoflurane anesthesia (Fig. 7a and Extended Data Fig. 9). To validate this observation beyond our measure of participation in cell assemblies, we plotted the mean firing rate of each of the three subpopulations over the sound stimulation and stimulation-free phases of our protocol before assembly identification. In line with its participation in sound-evoked assemblies in the awake state, the sound-specific population displayed increased activity during sound stimulation compared with the stimulus-free phases. Likewise, the ongoing assembly-specific population is more active in the stimulation-free period. When plotting the activity of the same subpopulations under isoflurane anesthesia, the modulations disappeared (Fig. 7b and Extended Data Fig. 9), validating the idea that single-cell properties are massively reassigned under anesthesia. Specificity to sound-evoked responses (although weaker) was still present under isoflurane anesthesia, but not necessarily in the same subgroups of neurons as in wakefulness (as seen when sorting cell properties in anesthesia; Fig. 7b).

To more precisely interpret the distribution of single-cell activity in wakefulness, we simulated three different hypotheses about the relationship between the probability of being recruited in a sound-evoked response and the probability of being recruited in an ongoing assembly. The first hypothesis that we considered was that evoked and ongoing probabilities are independent. To estimate the probability distribution that would result from this hypothesis, we reshuffled all probabilities across cells. This generated a much broader distribution of ongoing and evoked probabilities across cells than the one actually observed (Fig. 7c). This indicates that a correlation exists between the probability of engaging in sound-evoked responses and the probability of engaging in ongoing assemblies, in both the awake state and under anesthesia. The second hypothesis was that ongoing and evoked probabilities are equal and the observed distribution arises from probability estimation errors. This hypothesis led to a much narrower distribution of probabilities than observed in wakefulness but also under anesthesia (Fig. 7d versus Fig. 7a). The mean absolute distance of data points from the linear regression line fitted to spontaneous and evoked probabilities was 0.010 if we suppose equal recruitment probabilities (Fig. 7d), whereas it was 0.032 and 0.018 in the data for the awake and anesthetized states, respectively. The differences across all three measures were highly significant (*P* < 0.001, bootstrap test with 1,000 resamplings), indicating that, despite clear correlations between the probabilities of engaging in spontaneous and evoked events, neurons also tend to specialize for one or the other, and more so in the awake than in the anesthetized state.

In line with this, the data were much better approximated if we modeled the probabilities of engaging in a spontaneous or an evoked assembly as the sum of a common and independent probability, chosen randomly within continuous distributions (Fig. 7e). Altogether, this analysis shows, that under anesthesia, the recruitment of a cortical neuron in a sound response is more strongly determined by its probability of being recruited in a spontaneous event, leaving less freedom to encode sound information. In the awake state, the two probabilities are more dissociated, opening much larger possibilities to encode sound information.

## Discussion

Our data show that sensory inputs and ongoing activity engage distinct neuronal assemblies in the cortex during wakefulness and that during anesthesia sensory responses generate assemblies that also appear in ongoing activity. This observation reconciles contradictory earlier findings obtained in the two different states. In anesthetized animals, a recurrence of evoked responses in ongoing neuronal assemblies had been observed at mesoscopic and cellular resolutions^2,5^. This contrasted with recent reports in awake animals that ongoing activity is mostly orthogonal to evoked responses^10,11^. We provide, in the present study, a comparison of the spontaneous and evoked cell assemblies in both states, together with thalamic activity. We found that, although thalamic inputs to the cortex are distinct between spontaneous and evoked activity, under anesthesia sound stimuli engage stereotyped cortical cell assemblies that are already present in spontaneous activity. By contrast, sound stimuli evoke sound-specific cell assemblies in awake conditions, when sounds are perceived by the animal.

One main observation (Fig. 2) is that the cell assemblies evoked in the anesthetized cortex appear to engage cell assemblies already present in the spontaneous activity, where both spontaneous and evoked activities stem from the same restricted set of cortical cell assemblies, whereas much richer sets of assemblies are seen in the awake cortex. This restriction of the dynamics during anesthesia may explain previous findings that indicated a low dimensionality of sound responses in the anesthetized auditory cortex^23,32^. It is of interest that, if sensory information collapses under anesthesia (Fig. 4), it does not fully disappear, as indicated by sound classification performance clearly above chance levels and by the fact that almost half the neurons retain their response profile. This is in line with the common observation that stimulus-specific patterns still exist under anesthesia^19^. Although we could not directly assess this aspect, previous reports indicate that activity patterns in the anesthetized state follow well-known functional maps such as, for example, contour orientation maps in the visual cortex of carnivores^2,33^ or the tonotopic map in the auditory cortex^7,34^. These maps correspond to anatomically hardwired circuits^35,36^, which may constrain the spatial extent of the stereotypical population events that dominate cortical dynamics during anesthesia (Fig. 1). In humans, resting-state activity also displays reduced complexity and follows large-scale anatomical connectivity structures in the anesthetized state^37^. Our results further indicate that, while preserving these large-scale spatial features and the activity of a large number of individual neurons, anesthesia abolishes responses that carry more precise and more sparsely encoded information about the stimulus (Fig. 4). Comparisons of anesthetized and awake datasets from different experiments have suggested the absence of important features of auditory responses, such as offset^38^ or sustained responses^39,40^, in the cortex in the anesthetized state. We could directly show that, under anesthesia, many neurons change or lose the specific sound response properties that they had during wakefulness. Although it has been reported that pure-tone response properties are affected by anesthesia^19,41^, the use of diverse complex sounds in our study may have magnified the effects of anesthesia, by highlighting more diluted aspects of the auditory code^42^, which seem also to be more sensitive to anesthesia (Fig. 4). Along the same lines, the diversity of spontaneous responses may be reduced in anesthesia due to the absence of attentional modulations and motor or multisensory inputs that impact the auditory cortex^10,43,44^. Along with the larger diversity of sound responses, these important features of awake auditory cortical processing may contribute to the differences between spontaneous and evoked assemblies in the awake state.

Thanks to large-scale imaging at single-cell resolution, our results revealed a profound change in the cortical and thalamic population dynamics at a mesoscopic scale under anesthesia, whereas activity at the individual neuron level is partially preserved (Figs. 1–4). This indicates that anesthesia induces network-scale effects across cortical layers (Extended Data Fig. 3) and thalamocortical connections (Fig. 5). How anesthetics such as isoflurane, which potentiates inhibition^45^, lead to this profound change of dynamics remains a challenge for neural network modeling. The fact that, unlike the cortex, the thalamus generates different assemblies for ongoing and evoked activity under anesthesia indicates some degree of functional disconnection between the two structures, because the anesthetized cortex seems to ignore a difference that is present in its inputs. This indicates that the emergence of distinct cortical assemblies in awake conditions is of cortical origin.

The collapse of information through a dynamic change has been readily proposed as a mechanism for the loss of consciousness under anesthesia, along with a loss of functional connectivity^14,16,17^. Our results provide a strong quantitative support for this theory and suggest that the ability for the sensory cortex to form stimulus-specific neuronal assemblies that encode precise information about the sensory input could be a marker of wakefulness with respect to anesthesia. As sensory inputs evoke patterns present in spontaneous activity, one may assume that propagation of sensory information to other cortical areas will be limited, in agreement with findings in human anesthesia^46,47^. This may also explain the lost access to perception during anesthesia, and our results show that this loss already occurs in the primary sensory cortex. Finally, our results appear compatible with the idea that, in the awake cortex, the new assemblies formed by sensory inputs will propagate across the brain, but the mechanisms underlying this selective propagation are presently unknown, although previous models have proposed that asynchronous states may be the substrate of this selective propagation^48^.

## Methods

### Animals and surgery

We used C57BL/6 male and female 8- to 16-week-old mice. Animals were housed one to four animals per cage, in a normal light:dark cycle (12 h:12 h) in controlled humidity and temperature conditions (21–23 °C, 45–55% humidity). All procedures were in accordance with protocols approved by the French Ethical Committees nos. 59 and 89 (authorization no. 00275.01, APAFIS no. 9714-2018011108392486 v.2 and APAFIS no. 27040-2020090316536717 v.1).

Chronic window implantation surgery was performed in 4- to 6-week-old mice placed on a thermal blanket under anesthesia using a mix of ketamine (80 mg kg^−1^, Ketasol) and medetomidine (1 mg kg^−1^, Domitor, antagonized with atipamezole (Antisedan, OrionPharma) at the end of the surgery). The eyes were covered using Ocry gel (TVM Lab), and xylocaine 20 mg ml^−1^ (Aspen Pharma) was injected locally at the site where the incision was made. The right masseter was partially removed and a large craniotomy (~5-mm diameter) was performed above the auditory cortex using bone sutures of the skull as a landmark. For two-photon calcium imaging, we did 3–5 injections at 200-µm intervals of 150 nl (25 nl min^−1^) using pulled glass pipettes of rAAV1.syn.GCamP6s.WPRE virus (10^13^ virus particles per ml) diluted 30× (Vector Core). The craniotomy was then sealed with a 5-mm circular coverslip using cyanolit glue and dental cement (Ortho-Jet, Lang), and a metal post for head fixation was implanted and fixed with dental cement on the skull contralateral to the craniotomy.

For labeling of thalamocortical fibers, rAAV1.syn.GCamP6s.WPRE (10^13^ virus particles per ml) undiluted virus was stereotaxically injected into auditory thalamus (medial geniculate nucleus; anteroposterior = −3.0 mm, lateral = 2.1 mm, dorsoventral = 3.2). The fluorescence from thalamocortical fibers projecting into layer 1 of the auditory cortex could be recorded 4–5 weeks after injection.

### Two-photon imaging

For all isoflurane experiments, imaging was performed using a two-photon microscope (Femtonics) equipped with an 8-kHz resonant scanner combined with a pulsed laser (MaiTai-DS, SpectraPhysics) tuned at 900–920 nm using a ×10 objective (0.6 numerical aperture (NA), XLPLN10XSVMP, Olympus) immersed in ultrasound transmission gel (Aspet) previously centrifuged to eliminate air bubbles. Images were acquired at 31.5 Hz during blocks of 300 s using the Femtonics MESC 1.0 software. The imaging field of view was 1 × 1 mm^2^.

For KM and Zoletil experiments, imaging was performed using an acousto-optic, two-photon microscope (Kathala Systems) combined with a pulsed laser (Insight X3 Dual, SpectraPhysics) tuned at 920 nm using a ×16 objective (0.85 NA, Nikon) immersed in ultrasound transmission gel. The imaging field of view was 0.47 × 0.47 mm^2^, but four planes interspaced by 50 µm could be imaged simultaneously thanks to ultrafast defocusing with the acousto-optic deflectors. These four-plane stacks were imaged at 30.5 Hz during blocks of 150 s using the KIS 2.1 software from Karthala System.

Mice were habituated to stay head-fixed for 1 week 30–60 min per day before the recordings. Recordings were performed inside a light-tight, soundproof box. Imaging was performed at different depths ranging from 150 µm to 600 µm.

In five mice, after one awake imaging session, isoflurane anesthesia mixed in pure air was applied to the nose through a mask using the SomnoSuite anesthesia unit (Kent Scientific), without changing the field of view (see field-of-view stability in Fig. 1a,b). An infrared heat pad (Kent Scientific) was placed under the tube containing the mouse. Then, 3% of isoflurane was applied for 1 min to induce narcosis. Anesthesia was then slowly decreased until we observed whisker movements and a level close to 1.3% was applied during recordings (actual range 1.2–1.4%). Typically, the limit of narcosis was observed between 0.9% and 1.1% isoflurane concentration based on the occurrence of spontaneous whisker movements which were monitored with an infrared camera (Smartek Vision, objective Fujinon/25 mm). From this limit value, we increased concentration by 0.3%. Anesthesia was maintained at the same level during the 40 min of the imaging session.

KM or Zoletil (a 50:50 mix of tiletamine and zolazepam) was injected subcutaneously while leaving the animal under the microscope in head fixation to maintain an identical field of view. We applied a dose of 50 mg kg^−1^ of K and 1 mg kg^−1^ of M, which after an induction of 10 min was sufficient to maintain the animal stably anesthetized for more than 1 hour. After the imaging session, atipamezole was injected intramuscularly to accelerate waking up. Zoletil was applied at a dose of 70 mg kg^−1^ (that is, 35 mg kg^−1^ of tiletamine and 35 mg kg^−1^ of zolazepam). This dose was sufficient for maintenance of all three animals tested under anesthesia over 45 min.

Data collection and analysis were not performed blind to the conditions of the experiments. At a first level of screening, the mice with contaminated cranial windows and feeble labeling (low contrast or <200 neurons) were excluded from the experiments. At a second stage, during the preprocessing, the dataset with strong vertical motion artifacts, or feeble or absent auditory responses, were excluded.

### Stimulation protocol and sounds

The following sequence was applied in the awake state and then under anesthesia: two 300-s recordings without auditory stimulation, three 300-s recordings with sound stimulation, followed by two 300-s recordings without stimulations (Fig. 1a,b). Each sound was 500 ms long, and sound onsets and offsets were separated by a 1-s interval. All sounds were delivered at 192 kHz with a NI-PCI-6221 card (National Instrument) driven by Elphy2 (G. Sadoc, UNIC, France) through an amplifier and high-frequency loudspeakers (SA1 and MF1-S, Tucker-Davis Technologies). Sounds were calibrated in intensity at the location of the mouse ear using a probe microphone (Bruel & Kjaer).

During each of the 300-s stimulation sessions, each sound was played four times in a random order (in total, 12 presentations for each sound). We used a set of 50 predefined sounds divided into four groups (Extended Data Fig. 1): six frequency-modulated sounds (6–10, 10–16, 25–40 kHz, upward and downward modulations), ten complex sounds, six pure tones (4, 6, 10, 16, 25 and 40 kHz) and six amplitude-modulated sounds (sinusoidal modulation at 20, 7 and 3 Hz, for three carrier frequencies of 25 kHz and 4 kHz). All sounds (except for sinusoidal) were played at two intensities of 60 and 80 dB SPL (sound pressure level).

### Calcium signals processing and spike train estimation

Data analysis was performed using Matlab scripts. Motion artifacts were first corrected frame by frame, using a rigid body registration algorithm. A single set of ROIs corresponding to the neurons was defined by running Autocell (https://github.com/thomasdeneux/Autocell), a semiautomated hierarchical clustering algorithm based on pixel covariance over time^21^, on the concatenated data from the awake and anesthesia sessions. Neuropil contamination was subtracted^20^ by applying the following equation: *F*_corrected_(*t*) = *F*_measured_(*t*) – 0.7 *F*_neuropil_(*t*), where *F*_neuropil_(*t*) is estimated from the immediate surroundings (Gaussian smoothing kernel^3^, excluding the ROIs, *s* = 170 μm), where *F* is fluorescence and *t* time. The average neuropil signals shown in Extended Data Fig. 7 are computed by averaging *F*_neuropil_(*t*) across all putative neurons. We then applied the MLSpike deconvolution algorithm^22^ (github.com/Mlspike) to the neuropil-corrected raw fluorescent signal, which finds the most likely spike train underlying the recorded fluorescence using a maximum-likelihood approach taking into account baseline fluorescence fluctuations. The parameters used by the algorithm were the typical time constant of calcium transient (1.7 s) and a coefficient (range used: 4–6) adjusting for baseline drift compensation. Both these parameters were estimated to best fit the descending slope of experimental calcium spikes as well as the fluctuation of the baseline fluorescence. Time constant estimation was in accordance with the published estimations for GCaMP6s dynamics. After this process, every putative spike was described by its estimated onset time. We used the same spiking identification algorithm for the thalamocortical terminals with slightly different parameters (time constant 1.2 s and baseline drift compensation coefficient 6).

### Population events identification

After estimating spike trains with MLSpike (capable of estimating dense firing patterns (up to 20 Hz), where fluorescence rarely decays back to baseline), single-cell activity was described as a binary vector, where the number of elements was equal to the number of time frames during the recording (frame duration was 31 ms). A value of 1 was assigned at the time frames corresponding to the onset of each spike; otherwise, the vector was 0. The number of vectors was equal to the number of recorded neurons in the field of view, resulting in a matrix where columns corresponded to the time frames and rows to the neurons. Each column was summed to yield the number of neurons coactive at every time frame from which the instantaneous population firing rate could be deduced. As it is visible from sample raster plots (Fig. 1 and Extended Data Fig. 1), there were periods of time where spikes were much more synchronized across the population, which could be interpreted as population events, departing from the fluctuations of an asynchronous population spiking process. To identify (1) whether those peaks of activity were above asynchronous activity baseline and thus above coincidence by chance and (2) when exactly the periods of synchronization started and ended, we applied the following algorithm:

1. The order of interspike intervals of each neuron was independently reshuffled 100×, so that 100 surrogate matrices (number of neurons × number of time frames) were created. For each of these surrogate datasets, a population firing rate was calculated. A new matrix (100 × number of time frames) was created where each row is a population firing rate for each of the 100 reshuffling trials. From this matrix, we extracted the 99th percentile of the surrogate distribution within each time frame. The average 99th percentile across time frames was then calculated, and the final firing rate threshold was obtained by adding this average value to a local baseline estimate that aims to correct slow fluctuations of background firing rate. The baseline estimate was generated using an asymmetrical least squares smoothing algorithm with the parameters *p* = 0.01 for asymmetry and λ = 10^8^ for smoothness, adapted from Eilers and Boelens^26^.
2. Experimental population firing rate trace was smoothed using Savitzky–Golay filtering (with order 3 and frame length 7) in Matlab to get rid of nonessential peaks (Extended Data Fig. 1e, gray trace). Local maxima for the smoothed curve that were above the previously defined threshold were retained (red dots). The adjacent local minima around each of the local maxima were identified (blue and green circles). Time frames identified in this way were considered as the start (blue) and end (green) of population events (Extended Data Fig. 1e). All the neurons that had at least one spike during this time interval were defined as a neuronal assembly.
3. Neuronal assemblies were then described as binary vectors of length equal to the total number of neurons in the recorded field of view. A value of 1 indicated the participation of the cell in the assembly with at least one spike over its duration and a value of 0 indicated an absence of participation.

Neuronal assemblies could be detected in the same way during periods of stimulation and periods without stimulation (Extended Data Fig. 1e). However, in most of our analyses, neuronal responses evoked by sounds were quantified irrespective of the detection of a population event after sound onset. All neurons that emitted a putative spike during sound presentation (within a 500-ms interval from the start of the sound onset) were considered to belong to an evoked neuronal assembly. All population responses to sounds were taken into account (12 per sound) except if 0 spikes were detected across the entire population, as occasionally happened during anesthesia.

### Clustering of assemblies

The correlation matrix for ongoing assemblies and sound-evoked responses was constructed by computing Pearson’s correlation coefficient between binarized population vectors. When plotting the matrix, the colormap ranges from 0 to 1 unless otherwise stated. In Extended Data Fig. 1, assemblies are arranged in chronological order. In other figures, assemblies observed in the pre- and poststimulation blocks were separately reorganized using hierarchical clustering (agglomerative linkage clustering with furthest distance based on correlation metrics). Similar assemblies (that is, that shared a substantial number of neurons) were clustered together. For evoked responses, the organization was simply based on sound identity (except in Extended Data Fig. 2 where clustering was also applied to actually detected assemblies).

To quantify the reproducibility of same sound responses or ongoing assemblies within clusters, correlation was calculated across all sound repetitions or all assemblies of a cluster. Similarity across clusters and sound responses was calculated as the average correlation between all pairwise elements of the two compared groups of assemblies/responses. To measure maximal similarity between spontaneous and evoked activity, for each group of evoked responses, the spontaneous cluster with maximum crosscorrelation was identified.

For a given sound *i*, we estimate the similarity of population responses to ongoing population events as:

$$S_i = {\mathrm{Max}}\left\{ {C_{ij}} \right\}_j$$

where $C_{ij} = < r_{ij}(k,l) > _{k,l}$ and $r_{i_{}j}(k,l)$ is Pearson’s correlation coefficient between population vectors observed for presentation *k* (range 1–12) of sound *i* (range 1–50), and for assembly *l* from the ongoing assembly cluster number *j*. ${\mathrm{Max}}\left\{ {C_{ij}} \right\}_j$ is the maximum *C*_*ij*_ across all spontaneous clusters (that is, mean correlation with the ongoing event cluster *j*_*i*_ that is the most similar to sound *i*).

The similarity was compared with the mean internal reproducibility of responses to sound *i* and of assemblies within clusters *j*_*i*_:

$$R_i = (A_i + A_{j_i})/2$$

where $A_i = < r_i(k_1,k_2) > _{k_1 \ne k_2}$ is the average correlation across all distinct pairs of responses to sound *i* and $A_{j_i} = < r_{j_i}(l_1,l_2) > _{l_1 \ne l_2}$ is the average correlation across all distinct pairs of ongoing assemblies in cluster *j*_*i*_, which has maximal similarity with responses to sound *i*. Then, $r_i(k_1,k_2)$ (and, respectively, $r_{j_i}(l_1,l_2)$) is the correlation between distinct pairs of population vectors indexed by *k*_1_ and *k*_2_ (range 1–12) observed for sound *i* (respectively, indexed *l*_1_ and *l*_2_ for ongoing activity clusters *j*_*i*_). If sound-evoked assemblies are similar to a cluster of events occurring spontaneously, we expect *S*_*i*_ ≈ *R*_*i*_, otherwise *S*_*i*_ < *R*_*i*_.

### Dimensionality reduction

The neuronal state space is defined as the space of activity (probability of responding to a sound or participating in an ongoing assembly) of all simultaneously recorded neurons. A given event is represented by a vector that has the dimensions of the neuronal population. To represent population vectors in a three-dimensional (3D) space that captures a maximum of the variance without imposing nonlinear distortions of the space, we used principal component analysis (PCA). PCA was performed on a dataset pulling all ongoing assemblies and sound responses during both anesthesia and wakefulness. Projections of population vectors on two of the first three PCs were used for displaying them in a 2D space as in Fig. 3.

### Single-neuron quantifications and models

Lifetime sparseness was measured through the kurtosis of the response distribution^29^, a measure that is more general than other sparseness measures because it applies also to negative responses.

For both awake and anesthesia data, every neuron was characterized by a probability of responding to any sound (abscissa) and participating in any spontaneous population event (ordinate) (Extended Data Fig. 9a). The neurons plotted in this space were divided into three groups, separated based on the m.a.d. of the difference between evoked and spontaneous probabilities. All the neurons for which the probability difference was smaller than the mean probability difference + 1 m.a.d. and larger than the mean probability difference − 1 m.a.d. were classified as neurons with equal probability of responding to sounds or participating in a spontaneous event (Fig. 6a, green). Those with a probability difference below the mean probability difference − 1 m.a.d. (Fig. 6a, red) were classified as having a higher probability of responding to sounds, and those with a difference above the mean probability difference + 1 m.a.d. (Fig. 6a, blue) were classified as having a higher probability of participating in spontaneous events. m.a.d. was chosen instead of s.d. because the probability difference was not normally distributed.

To better capture how responsiveness to ongoing and evoked events was distributed across the population, we defined three different probability models. The first model simply assumes that the probability of being active in an ongoing event is independent of the probability of being active in an evoked response. The expected distribution of probabilities for this hypothesis was generated by randomly shuffling observed probabilities across cells, generating a distribution that is much broader than the one observed for our data. The second model assumes that the probability of being active in either an ongoing event or a sound response is identical. This model was simulated by first defining the distribution of probabilities of participating in an ongoing event for 6,310 surrogate neurons. The average probability is set to 0.11 ± 0.08, in accordance with experimental data. Identical values were attributed to the probabilities of responding to any sound.

To more closely simulate the wakefulness condition, the last model supposes that, for every neuron *i*, the probability of response to a sound is *p*_resp_(*i*) *=* *p*_common_(*i*) + *p*_sound___spec_(*i*), and the probability of participating in an ongoing event is *p*_ongoing_(*i*) *=* *p*_common_(*i*) + *p*_spont___spec_(*i*). The value of *p*_common_(*i*) is drawn from a random distribution exp(−(*x/k*)^2^)/*S* for *x* between 0 and 1. *S* is the integral of exp(−(*x*/*k*)^2^) between 0 and 1; *p*_*s*ound___spec_(*i*) and *p*_spont___spec_(*i*) are drawn from two independent Gaussian distributions centered on 0 and of variance ${v^2}_{{\mathrm{sound}}}$ and ${v^2}_{{\mathrm{spont}}}$. The parameters were chosen to fit the experimental distribution of the difference in probabilities: *k* = 0.1, ${v}_{{\mathrm{sound}}}$ = 0.85 and ${v}_{{\mathrm{spont}}}$ = 0.65.

### Template-matching classifier

To quantify the sound specificity of the patterns of neuronal assemblies, we used a crossvalidated template-matching algorithm, where correlation was the metric between population vectors. We used a leave-one-out crossvalidation procedure with a training set of eleven sound presentations and a test set of one sound presentation. This was repeated 12×, changing the test sound presentation each time. At every iteration of classification, the response to the test sound presentation was compared with the 50 × 12 − 1 other single trial sound responses using the correlation distance as a metric. The test response was then attributed to the sound that has the smallest average distance with its single trial responses (excluding the test response in the calculation).

### Clustering of single neurons

Clustering was also used to organize neurons according to the similarity of their responses to the sounds. Due to the large variability observed in many neurons, this analysis is not exhaustive but rather aims at identifying principal classes of responses within our dataset. Clustering was performed across the five cortical imaging sessions, thus including 3,641 neurons, and the seven thalamic axon imaging sessions (13,314 terminals), in both awake and anesthetized states. Each neuron was characterized by a vector of 50 elements (corresponding to the number of presented sounds), where each element contained the information about the number of responses during the trial (from 0 to 12). Pearson’s correlation matrix for all neuron/terminal response vectors was constructed by computing Pearson’s correlation coefficient between them. Then neurons/terminals were reorganized using hierarchical clustering (agglomerative linkage clustering with the furthest distance algorithm, based on correlation metrics). The groups of neurons sharing similar sound response profiles were assessed. This method yielded a number of strongly correlated clusters of neurons that were tuned to multiple sounds, few clusters specific to a single sound and several small clusters, which after visual inspection appeared to contain noisy responses (hence very dissimilar to other clusters). Several of the clusters of thalamocortical terminals were specific to single sounds. In the case of thalamocortical terminals, due to the response sparseness across the large population of putative axonal terminals, only the ones that responded to any sound at least twice were taken into consideration before clustering (representing 25% in the awake state and 5% under anesthesia). To identify the sounds to which every cluster was significantly tuned in different conditions (awake and anesthesia), the sound responses of every neuron within a given cluster were reshuffled over all the stimulation instances (597). The number of nonzero reshuffled responses for 12 randomly chosen instances (out of 597, corresponding to the number of presentations of the same sound during the trial) was averaged over all neurons of the cluster. We used one-way analysis of variance (ANOVA; Matlab, Mathworks), separately applied to every cluster and every condition (awake, anesthesia), to identify the sounds that had significantly higher responses than expected from the shuffled dataset. The ANOVA function also returns the particular sounds for which the response was significant.

### Statistical analysis

All statistical analyses were performed using built-in MATLAB functions. The following tests were used: one-way ANOVA followed by Tukey’s multiple comparison test (anova1 and multcompare), Wilcoxon’s rank-sum test (two sided, the equivalent of the Mann–Whitney *U*-test) and paired Wilcoxon’s signed-rank test (two sided). No statistical methods were used to predetermine sample sizes, but our sample sizes are similar to those reported in previous publications^10,28,30^. All tests except ANOVA were nonparametric without assumption on the data distribution. For the ANOVA, data distribution was assumed to be normal, but this was not formally tested.

### Reporting summary

Further information on research design is available in the Nature Research Reporting Summary linked to this article.

## Online content

Any methods, additional references, Nature Research reporting summaries, source data, extended data, supplementary information, acknowledgements, peer review information; details of author contributions and competing interests; and statements of data and code availability are available at 10.1038/s41593-022-01168-5.

## Supplementary information

Reporting summary

## Data Availability

All data are freely available on *Zenodo*^49^ (10.5281/zenodo.6802671). Source data are provided with this paper.

## Source data

Source Data Fig. 1 Statistical source data.
Source Data Fig. 2 Statistical source data.
Source Data Fig. 4 Statistical source data.
Source Data Fig. 5 Statistical source data.
Source Data Fig. 6 Statistical source data.
Source Data Extended Data Fig. 6 Statistical source data.

## Extended data

are available for this paper at 10.1038/s41593-022-01168-5.

## Code availability

All customized code used in the analysis is freely available on *Zenodo*^49^ (10.5281/zenodo.6802671). A Matlab toolbox with customized functions used in the present study is also available on GitHub: https://github.com/Einwohner/Neuronal-assemblies-analysis.
