# Supplementary material for: Awake perception is associated with dedicated neuronal assemblies in the cerebral cortex
Source: Nat Neurosci. 2022 Sep 28;25(10):1327–38. doi: 10.1038/s41593-022-01168-5 (PMC9534770; doi:10.1038/s41593-022-01168-5)
Supplement: Supplementary file 1 — Reporting summary [file 41593_2022_1168_MOESM1_ESM.pdf]

nature portfolio

NN-A77277
Corresponding author(s): Brice Bathellier, Alain Destexhe
Last updated by author(s): 16/8/2022

Reporting Summary

Nature Portfolio wishes to improve the reproducibility of the work that we publish. This form provides structure for consistency and transparency in reporting. For further information on Nature Portfolio policies, see our Editorial Policies and the Editorial Policy Checklist.

Statistics

For all statistical analyses, confirm that the following items are present in the figure legend, table legend, main text, or Methods section.

n/a	Confirmed	
☐	☒	The exact sample size (*n*) for each experimental group/condition, given as a discrete number and unit of measurement
☐	☒	A statement on whether measurements were taken from distinct samples or whether the same sample was measured repeatedly
☐	☒	The statistical test(s) used AND whether they are one- or two-sided<br>*Only common tests should be described solely by name; describe more complex techniques in the Methods section.*
☐	☒	A description of all covariates tested
☐	☒	A description of any assumptions or corrections, such as tests of normality and adjustment for multiple comparisons
☐	☒	A full description of the statistical parameters including central tendency (e.g. means) or other basic estimates (e.g. regression coefficient) AND variation (e.g. standard deviation) or associated estimates of uncertainty (e.g. confidence intervals)
☐	☒	For null hypothesis testing, the test statistic (e.g. *F*, *t*, *r*) with confidence intervals, effect sizes, degrees of freedom and *P* value noted<br>*Give P values as exact values whenever suitable.*
☒	☐	For Bayesian analysis, information on the choice of priors and Markov chain Monte Carlo settings
☐	☒	For hierarchical and complex designs, identification of the appropriate level for tests and full reporting of outcomes
☐	☒	Estimates of effect sizes (e.g. Cohen's *d*, Pearson's *r*), indicating how they were calculated

Our web collection on statistics for biologists contains articles on many of the points above.

Software and code

Policy information about availability of computer code

Data collection	Elphy2 (G. Sadoc, UNIC, France), Mesc 1.0 acquisition software (Femtonics, Budapest, Hungary), KIS 2.1 Acquisition software (Karthala, Paris, FR)
Data analysis	Autocell (https://github.com/thomasdeneux/Autocell), MLSpike algorithm (github.com/Mlspike), custom MatLab codes for assemblies identification and analysis (https://github.com/Einwohner/Neuronal-assemblies-analysis). All custom code used in the analysis is freely available on Zenodo49 https://doi.org/10.5281/zenodo.6802671

For manuscripts utilizing custom algorithms or software that are central to the research but not yet described in published literature, software must be made available to editors and reviewers. We strongly encourage code deposition in a community repository (e.g. GitHub). See the Nature Portfolio guidelines for submitting code & software for further information.

Data

Policy information about availability of data

All manuscripts must include a data availability statement. This statement should provide the following information, where applicable:

- Accession codes, unique identifiers, or web links for publicly available datasets
- A description of any restrictions on data availability
- For clinical datasets or third party data, please ensure that the statement adheres to our policy

All data is freely available on Zenodo https://doi.org/10.5281/zenodo.6802671.

Human research participants

Policy information about studies involving human research participants and Sex and Gender in Research.

Reporting on sex and gender	not applicable
Population characteristics	not applicable
Recruitment	not applicable
Ethics oversight	not applicable

Note that full information on the approval of the study protocol must also be provided in the manuscript.

Field-specific reporting

Please select the one below that is the best fit for your research. If you are not sure, read the appropriate sections before making your selection.

☒ Life sciences ☐ Behavioural & social sciences ☐ Ecological, evolutionary & environmental sciences

For a reference copy of the document with all sections, see nature.com/documents/nr-reporting-summary-flat.pdf

Life sciences study design

All studies must disclose on these points even when the disclosure is negative.

Sample size	For imaging experiments, between 5 and 11 mice were imaged per experimental paradigm; the number of mice was dependent on the yield of usable mice and the number of animals initially implanted and injected. Altogether, 19 animals were recorded in the study. Cortical neurons were recorded in 12 mice (20 were injected with AAV1 GCaMp6s in the ACx, 8 were discarded because of bad quality of labeling or window contamination). 11 mice were recorded in awake state, 5 out of them were recorded both in awake and anesthetized states and one mouse was recorded only at anesthetized state (awake state was not exploitable because of strong movement artifacts). Thalamo-cotrical fibers were recorded in 7 mice (8 were injected in MGV) both in awake and anesthetized states. One trial was chosen for each mouse based on sound responsiveness and movement artifact. The sample size was 300-1200 cortical neurons per mouse. Sample size for imaging experiments was determined by the current standard used for mice in neuroscience, based on the minimal amount of mice required to detect significance with an alpha rate set at 0.05 in a standardly powered experiment.
Data exclusions	At the first level of screening the mice with contaminated cranial windows and feeble labeling (low contrast or less than 200 neurons) were excluded from the consideration. At the second stage, during the preprocessing, the trials with strong motion artifacts (in Z) or feeble or absent auditory response were excluded.
Replication	Each experiment presented in the paper was repeated in multiple animals (between 5 and 11 per condition). A few animals were excluded from the analysis but only due to poor cranial window preparation or weak GCAMP6s labeling. All results in the paper are drawn from the analysis of multiple animals. Importantly, every sound out of 50 sounds was presented 12 times and the patterns of response were reproducible in all recorded mice (see confirmation in Extended data Fig 3a) using a field of view of 1x1mm. The pattern of auditory responses in thalamo-cortical fibers was more variable because of the smaller field of view (300x300 um) imposed by the smaller size of the terminals. So only a fraction of auditory cortex could be recorded at a time. All experiments were successfully replicated.
Randomization	Animals were assigned randomly to the experimental groups
Blinding	The investigators were not blinded during data collection. Blinding was not relevant to this study because all key results do not come from a a comparison of different experimental groups but from the comparison of two conditions (awake vs anesthesia) in the same animals. Computational analysis was not performed blinded because the same exact analysis script was applied to the two compared conditions (anesthesia vs awake).

Reporting for specific materials, systems and methods

We require information from authors about some types of materials, experimental systems and methods used in many studies. Here, indicate whether each material, system or method listed is relevant to your study. If you are not sure if a list item applies to your research, read the appropriate section before selecting a response.

Materials & experimental systems

n/a	Involved in the study	
☒	☐	Antibodies
☒	☐	Eukaryotic cell lines
☒	☐	Palaeontology and archaeology
☐	☒	Animals and other organisms
☒	☐	Clinical data
☒	☐	Dual use research of concern

Methods

n/a	Involved in the study	
☒	☐	ChIP-seq
☒	☐	Flow cytometry
☒	☐	MRI-based neuroimaging

Animals and other research organisms

Policy information about studies involving animals; ARRIVE guidelines recommended for reporting animal research, and Sex and Gender in Research

Laboratory animals	C57bl6 male and female 8-16 weeks mice. Animals were housed 1–4 animals per cage, in a normal light/dark cycle (12 h/12 h) in controlled humidity and temperature conditions (21-23°C, 45-55% humidity).
Wild animals	No wild animals were used in this study.
Reporting on sex	No sex-based analysis was performed. We used C57bl6, 8-16 weeks mice, 7 male and 7 females.
Field-collected samples	No field collected samples were used in this study.
Ethics oversight	All procedures were in accordance with protocols approved by the French Ethical Committees #59 and #89 (authorizations 00275.01, and APAFIS#9714-2018011108392486 v2 and APAFIS#27040-2020090316536717 v1).

Note that full information on the approval of the study protocol must also be provided in the manuscript.
